# Mutations in a barley cytochrome P450 gene enhances pathogen induced programmed cell death and cutin layer instability

**DOI:** 10.1371/journal.pgen.1009473

**Published:** 2021-12-16

**Authors:** Gazala Ameen, Shyam Solanki, Lauren Sager-Bittara, Jonathan Richards, Prabin Tamang, Timothy L. Friesen, Robert S. Brueggeman

**Affiliations:** 1 Department of Agronomy, Horticulture & Plant Science, South Dakota State University, Brookings, South Dakota, United States of America; 2 Department of Plant Pathology, North Dakota State University, Fargo, North Dakota, United States of America; 3 Department of Plant Pathology and Crop Physiology, Louisiana State University Agricultural Center, Baton Rouge, Louisiana, United States of America; 4 USDA-ARS, Natural Products Utilization Research Unit, Oxford, Mississippi, United States of America; 5 USDA-ARS, Red River Valley Agricultural Research Center, Cereal Crops Research Unit, Fargo, North Dakota, United States of America; 6 Department of Crop and Soil Sciences, Washington State University, Pullman, Washington, United States of America; University of California Davis, UNITED STATES

## Abstract

Disease lesion mimic mutants (DLMMs) are characterized by the spontaneous development of necrotic spots with various phenotypes designated as necrotic (*nec*) mutants in barley. The *nec* mutants were traditionally considered to have aberrant regulation of programmed cell death (PCD) pathways, which have roles in plant immunity and development. Most barley *nec3* mutants express cream to orange necrotic lesions contrasting them from typical spontaneous DLMMs that develop dark pigmented lesions indicative of serotonin/phenolics deposition. Barley *nec3* mutants grown under sterile conditions did not exhibit necrotic phenotypes until inoculated with adapted pathogens, suggesting that they are not typical DLMMs. The F_2_ progeny of a cross between *nec3-*γ1 and variety Quest segregated as a single recessive susceptibility gene post-inoculation with *Bipolaris sorokiniana*, the causal agent of the disease spot blotch. *Nec3* was genetically delimited to 0.14 cM representing 16.5 megabases of physical sequence containing 149 annotated high confidence genes. RNAseq and comparative analysis of the wild type and five independent *nec3* mutants identified a single candidate cytochrome P450 gene (HORVU.MOREX.r2.6HG0460850) that was validated as *nec3* by independent mutations that result in predicted nonfunctional proteins. Histology studies determined that *nec3* mutants had an unstable cutin layer that disrupted normal *Bipolaris sorokiniana* germ tube development.

## Author summary

At the site of pathogen infection, plant defense mechanisms rely on controlled programmed cell death (PCD) to sequester biotrophic pathogens that require living cells to extract nutrients from the host. However, these defense mechanisms are hijacked by necrotrophic plant pathogens that purposefully induce PCD to feed on the dead cells, thus facilitating further disease development. Thus, understanding PCD responses is important for resistance to both classes of pathogens. We characterized five independent disease lesion mimic mutants of barley designated necrotic 3 (*nec3*) that show aberrant regulation of PCD responses upon pathogen challenge. A cytochrome P450 gene was identified as *Nec3* encoding a Tryptamine 5-Hydroxylase that functions as a terminal serotonin biosynthetic enzyme in the Tryptophan pathway of plants. We posit that *nec3* mutants have disrupted serotonin biosynthesis resulting in expanded PCD, necrotrophic pathogen susceptibility and cutin layer instability. The *nec3* mutants show expanded PCD and disease susceptibility of pathogen-induced necrotic lesions, suggesting a role of serotonin to sequester PCD and suppress pathogen colonization. The identification of *Nec3* will facilitate functional analysis to elucidate the role that serotonin plays in the elicitation or suppression of PCD immunity responses to diverse pathogens and the effects it has on cutin layer biosynthesis.

## Introduction

Programmed cell death (PCD) is a highly evolved and tightly regulated physiological response of plant and animal cells that plays a role in development, cell differentiation, cell number homeostasis, and immunity [1]. In plants, PCD is activated by environmental cues, including biotic stress-induced by pathogens and represents the major physiological response and mechanism of defense against invading microbes or feeding invertebrates. Specialized or adapted plant pathogens evolved to produce and utilize virulence effectors to facilitate host penetration and colonization by manipulating the host cellular machinery to induce inappropriate physiological responses that promote access to nutrients and life cycle completion [2].

The plant’s innate immune system evolves transmembrane receptors known as pattern recognition receptors (PRRs) that detect invading pathogens in the apoplastic space as the first line of defense and is known as pathogen-associated molecular patterns (PAMP) triggered immunity (PTI). The PTI responses activate underlying cytosolic signaling cascades, including the mitogen-activated protein kinase (MAPK) pathways [3] inducing callose deposition at the point of pathogen penetration, expression of pathogenesis-related (PR) genes, a quick transient reactive oxygen species (ROS), hypersensitive response (HR), and in most responses the resulting necrotic lesions at the site of attempted pathogen penetration is accompanied by the deposition of phenolic compounds. The selective pressures exerted by pathogens forced plant innate immune systems to counter-evolve to recognize pathogen virulence effectors [4–7] and more commonly their manipulation of targeted host proteins [8]. A well-characterized example is the *Pseudomonas syringae* effectors that inhibit FLS2 PRR-mediated signaling following flg22 perception [9]. For biotrophic pathogens, that require living host cells to feed, these effectors no longer facilitate colonization but rather alert the host to their presence, eliciting PCD that kills the cells they are feeding on, effectively stopping the colonization process. Thus, HR is critical to plant innate immunity against biotrophic plant pathogens, including viruses, bacteria, fungi, oomycetes, and invertebrates [10]. However, the necrotrophic pathogens that acquire nutrients from dying cells such as *Parastagonospora nodorum* [11] and *Pyrenophora teres* [12] have adapted to hijack these gene-for-gene immunity mechanisms by evolving necrotrophic effectors (NEs) that purposely alert the host immune system of their presence through immunity receptor activation. These inverse-gene-for-gene interactions [13] initiate PCD responses, which the necrotrophic pathogens utilize to facilitate disease formation because they acquire nutrients from the resulting dying and dead tissues. Thus, necrotrophic pathogens can complete their lifecycle on the host by facilitating further disease development through necrotrophic effector-triggered susceptibility (NETS); [12]. Both biotrophic and necrotrophic pathogens elicit PCD immunity responses in plants with different outcomes, incompatibility–vs- compatibility, respectively, determined by the lifestyle of the pathogen and the timing of the responses. Thus, knowledge of PCD pathways is important to understand resistance and susceptibility mechanisms in crop plants when interacting with both classes of pathogens.

The disease lesion mimic mutants (DLMMs) that spontaneously produce PCD are important resources to decipher the regulation of the cell death pathways [14]. However, very few DLMMs have been thoroughly characterized. In barley, several DLMMs have been described [14], but only two DLMM genes have been identified, *Hvnec1* and *mlo*. The *Hvnec1* gene encodes a cyclic-gated ion channel protein [15,16] with sequence homology to the *Arabidopsis thaliana HLM1* gene [17]. Like *HLM1*, *Hvnec1* has increased pathogenesis-related (PR) protein expression and produces spontaneous necrotic lesions and leaf tip necrosis with increased susceptibility to certain pathogens [18,19]. The *mlo* gene, which confers increased resistance to the fungal pathogen *Erysiphe graminis* f. sp. *hordei*, the causal agent of powdery mildew, has been deployed in Northern Europe and has served as a source of durable resistance in barley for 37 years [20]. However, in the absence of the disease, *mlo* causes ~4% reduction in yield due to its DLMM phenotype, which results in loss of photosynthetic potential and thus is only economically advantageous under high disease pressure [21]. The second cost of *mlo* deployment is enhanced susceptibility to several necrotrophic pathogens, including *Bipolaris sorokiniana* the causal agent of the barley disease spot blotch [22], *Fusarium graminearum* the cause of fusarium head blight [23], *Ramularia collo-cygni* the causal agent of Ramularia leaf spot [24] and *Magnaporthe oryzae* the causal agent of the rice blast disease [25]. The *Mlo* gene encodes a ROP like G-protein that appears to be a suppressor of PCD and is conserved and found in other species, including pea, *Arabidopsis* and tomato [25].

**Fig 1:**
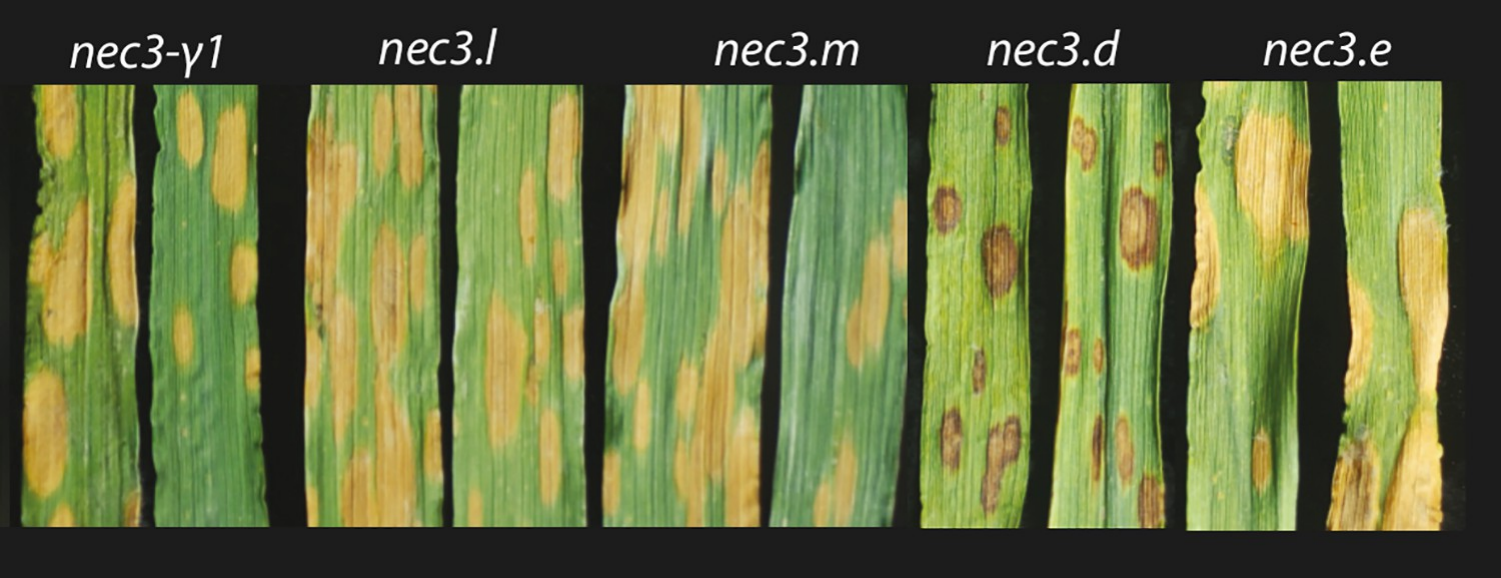
Typical phenotypic reactions of the *nec3* mutants. The *nec3* mutants are shown from left to right (*nec3-γ1*, *nec3*.l, *nec3*.m, *nec3*.d and *nec3*.e) after infection with *Bipolaris sorokiniana* isolate ND85F.

In barley, both chemical and irradiation mutagenesis have been utilized to induce a large collection of DLMMs [26]. One DLMM mutant was designated as *nec3* and was shown to produce distinct cream to orange necrotic lesions (Fig 1). The *nec3-γ1* mutant described here was originally identified from γ-irradiated M_2_ seedlings in an attempt to identify barley mutants resistant to *B*. *sorokiniana* isolate ND90Pr. This study was initiated to identify mutants in a putative cv Bowman dominant susceptibility factor [27]. The previously identified *nec3* mutants were irradiated using x-ray mutagenesis in the cv Proctor and Villa backgrounds and by fast neutron mutagenesis in the cv Steptoe background [28]. The *nec3*.*d* (GSHO 1330) mutant was generated in cv Proctor (PI 280420) and the *nec3*.*e* (GSHO 2423) mutant was generated in cv Villa (PI 399506). The *nec3*.*l* (GSHO 3605 formally known as FN362) and *nec3*.*m* (GSHO 3606 formally known as FN363) mutants were generated in the cv Steptoe (CIho 15229) background. The *nec3*.*d* and *nec3*.*e* mutants used in this study were near isogenic lines developed by recurrent backcrossing into the cv Bowman background. Four of the five allelic *nec3* mutants express a distinctive programmed cell death phenotype with tan to orange necrotic lesions without the dark phenolics usually associated with DLMMs. The previous attempt to identify *nec3* utilizing morphological markers localized *nec3* to the centromeric region of barley chromosome 6HS ~29.2 cM distal of the *rob1* (orange lemma 1) locus [26,29,30].

In this study genetic mapping and RNAseq-based gene structure identification among the WT and mutants identified the candidate *Nec3* gene (HORVU.MOREX.r2.6HG0460850), which was predicted to encode a Cytochrome P450, designated as *HvCYP71-A1*. Allele analysis of five independent *nec3* mutants identified five different mutations that were detrimental to *HvCYP71-A1* function, validating it as *Nec3*. We report that *Nec3* putatively encodes a tryptamine 5-hydroxylase, which catalyzes the conversion of tryptamine to serotonin, similar to the rice lesion mimic phenotype producing *SL* gene [31,32]. In addition, we show that the *nec3* phenotype is not a spontaneous DLMM but is rather induced by several species of *Ascomycete* pathogenic fungi, both necrotrophs and biotrophs and the bacterial pathogen *Xanthomonas translucens*, representing pathogens that penetrate the host cells directly by disrupting the cell wall and plasma membrane. We also determined that the *nec3* mutants have an unstable cutin layer that possibly peels away from the leaf surface when in contact with the pathogen *Bipolaris sorokinina* germ tubes. This aberrant interaction possibly alters the pathogen’s developmental signaling resulting in profuse branching of the fungal germ tubes on the mutant leaf surface. Our study will facilitate further *Nec3* functional analysis and the roles it plays in the elicitation or suppression of PCD responses to diverse pathogens and the effects it has on cutin layer biosynthesis.

## Results

### Pathogen induction of the nec3 phenotype

The *nec3* lesions consistently occurred on the five independent *nec3* mutants under normal greenhouse conditions even without pathogen challenge and were presumed to be lesion mimic mutants with spontaneous lesion development. To test the hypothesis that the *nec3* lesions were spontaneous, the wildtype and mutant plants were grown in a sterile isolation box. These plants did not express the *nec3* phenotype and grew to the adult plant stage (Feekes 10.5; full head emergence) without showing any necrotic lesions. The non-inoculated *nec3* seedlings grown on the greenhouse bench outside the isolation box exhibited the *nec3* phenotype at the second leaf stage and continued to develop these distinctive lesions (Fig 1) through the adult plant stages. Histological characterization of the fungal structures that grew from the lesions of the non-inoculated *nec3* plants showed that they were primarily colonized by *Blumeria graminis*, which is endemic in the greenhouses at Washington State University and North Dakota State University, where these experiments were conducted.

**Fig 2:**
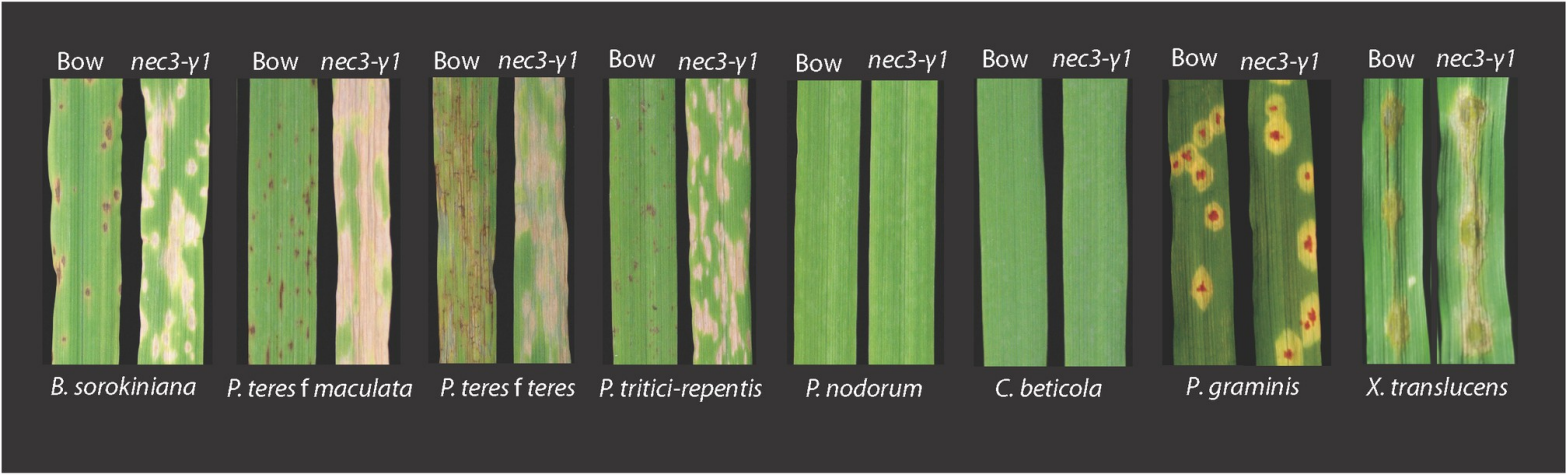
The *nec3* phenotype was induced by *Bipolaris sorokiniana*, *Pyrenophora teres* f. *teres*, *Pyrenophora teres* f. *maculata*, *Pyrenophora tritici repentis*, and *Xanthomonas translucens* pv *undulosa*. Each panel shows the typical reaction to the pathogen labelled below with wildtype Bowman (Bow) on the left and the *nec3- γ1* mutant on the right as labeled above. Inoculations with *Puccinia graminis* f. sp. *tritici race QCCJB*, *Cercospora beticola* and *Parastagonospora nodorum* did not induce the *nec3* phenotype.

To determine if the pathogen-induced *nec3* phenotypes were dependent on pathogen infection, we challenged wildtype and mutant plants with several different pathogens with both virulent and avirulent responses on wildtype Bowman or Steptoe plants. Inoculations performed under sterile environmental conditions in growth chambers determined that the typical *nec3* lesions were elicited on *nec3-γ1* mutant seedlings by the necrotrophic ascomycete fungal pathogens *Bipolaris sorokiniana*, *Pyrenophora teres* f. *teres*, *Pyrenophora teres* f. *maculata*, and *Pyrenophora tritici repentis*, as well as the biotrophic ascomycete fungal pathogen *Blumeria graminis* (Fig 2; *Blumeria graminis* inoculated plants are not shown). Both the virulent and avirulent isolates of these pathogens produced the expected susceptible or resistant response on wildtype Bowman or Steptoe but induced the typical *nec3* lesions on the *nec3-γ1* mutant. Infiltration inoculations with the bacterial pathogen *Xanthomonas translucens* pv *undulosa*, which resulted in a resistant reaction on Bowman wildtype, also induced the *nec3* phenotype on the *nec3-γ1* mutant (Fig 2). The *nec3* phenotype was not induced when *nec3-γ1* mutant seedlings were inoculated with the basidiomycete biotrophic fungal pathogen *Puccinia graminis* f. sp. *tritici* race QCCJB that is virulent on cv Bowman, which carries the *Rpg1* stem rust resistance gene (Fig 2). The *nec3* phenotype was also not induced when *nec3-γ1* mutant seedlings were inoculated with *P*. *graminis* f. sp. *tritici* race HKHJC that is avirulent on cv Bowman due to resistance provided by *Rpg1* (Picture not shown). The barley non-host necrotrophic pathogens *Cercospora beticola* and *Parastagonospora nodorum* did not induces the *nec3* phenotype.

### Allelism crosses

**Fig 3:**
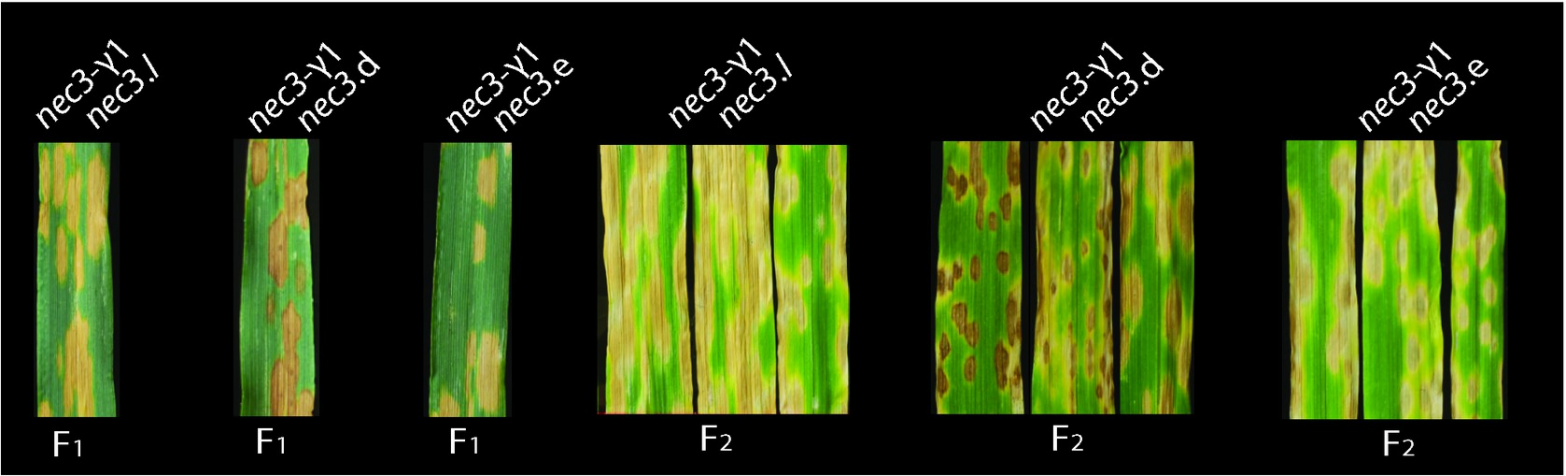
The typical phenotypes of the F_1_ and F_2_ progeny of *nec3* allelism tests. The first three panels show the phenotype of F_1_ progeny of the *nec3* mutants from allelic test crosses after infection with *Bipolaris sorokiniana* isolate ND85F. The progeny from the allelism test crosses were advanced to the F_2_ generation and assayed for the *nec3* phenotype post *B*. *sorokiniana* isolate ND85F inoculation. The allelism test crosses are shown above and generations shown below.

Allelism crosses were performed in the field to determine if the four confirmed *nec3* mutants, *nec3*.d, *nec3*.e, *nec3*.l, and *nec3*.m were allelic to *nec3-γ1*. Crosses between *nec3-γ1* and the mutants, *nec3*.*d*, *nec3*.*e* and *nec3*.*l e* were successful but due to the head sterility effect of the *nec3* mutants, only 2–5 F_1_ seeds were planted from each allelism test cross. 100% of the F_1_ plants displayed the *nec3* phenotype following inoculation with *B*. *sorokiniana* isolate ND85F under greenhouse conditions (Fig 3). The isolate ND85F of *B*. *sorokiniana* used in this study is pathotype 1 (Steptoe and Proctor are susceptible and Bowman and Villa are resistant) which has been widely used in spot blotch association and bi-parental mapping. The F_2_ seed of each cross was planted in the field in rows containing 20 individual F_2_ plants adjacent to a spot blotch disease nursery containing spreader rows inoculated with *B*. *sorokiniana* isolate ND85F. 100% of the F_2_ individuals exhibited the *nec3* phenotype (Fig 3) demonstrating that the *nec3*-γ1 mutation was allelic to the other known *nec3* mutants. Interestingly, the allelism cross (*nec3*.*d* x *nec3*-γ1) with the *nec3*.*d* mutant, which expresses dark lesions typical of phenolics build up and are smaller in size compared to the other *nec3* mutants, produced F_2_ progeny that expressed a range of lesion phenotypes, from the typical *nec3*.*d* to the *nec3*. *γ1* phenotype and a blending of both (Fig 3). These results suggested that the segregation of the two *nec3* alleles had a blending effect on the lesion phenotypes.

### Culture filtrate infiltration

A culture infiltration experiment was used to determine if the nec3 elicitor was present and if it is proteinaceous. The WT Bowman (resistant to isolate ND85F) and WT Steptoe (susceptible to isolate ND85F) and the *nec3* mutants *nec3-γ1*, *nec3*.*d* and *nec3*.*e* in the Bowman background and *nec3*.*l* and *nec3*.*m* in the Steptoe background showed differential reactions after infiltration with *B*. *sorokiniana* isolate ND85F culture filtrates and controls infiltrated into secondary leaves (S1 Fig). The infiltrations containing culture filtrates + Fries Media, culture filtrates + MOPS buffer, and culture filtrates + MOPS + pronase on the mutants *nec3-γ1*, *nec3*.*d*, *nec3*.*e*, *nec3*.*l* and *nec3*.*m* showed typical *nec3* PCD as previously described with a lack of defined margins around the lesions (S1 Fig). The control infiltrations containing Fries Media + MOPS buffer + pronase did not induce a reaction on the mutants or WT leaves. The WT Bowman leaves showed no response to any of the infiltrations, culture filtrates or control. However, the WT Steptoe seedlings displayed a PCD response to all the infiltrations containing culture filtrates that were different than *nec3*.*l* and *nec3*.*m*, the *nec3* mutants in the Steptoe background. Steptoe reacted to the culture filtrates by forming necrotic lesions with dark margins and a dark necrotic center, which resembled susceptible lesions that are typically induced by the necrotrophic pathogen *B*. *sorokiniana* that contain phenolics accumulation.

### Exogenous supplementation of DAMPs, and PAMPs and supplementation with Serotonin and Tryptamine

To determine if known DAMPs or PAMPs elicit the *nec3* phenotype infiltrations with a known DAMP and PAMPs was performed. Infiltrations of WT Bowman, *nec3-γ1*, WT Steptoe and *nec3*.*l* with the known DAMP trigalacturonic acid was carried out at three concentrations (10mg/mL, 1mg/mL and 0.1mg/mL); the bacterial PAMP FLG22 at a concentration of 1mg/mL and chitin (C9752, Sigma-aldrich) (100μg/ml) resulted in no observable reactions up to one week after infiltration (S2 Fig), as compared to the mock controls treated with water. To determine if Serotonin or Tryptamine accumulation effects the *nec3* phenotype an exogenous root feeding experiment was conducted. Exogenous root feeding of Serotonin and Tryptamine did produce visible phenotypic variance in the size and shape of *nec3* lesions in *nec3-γ1*, *nec3*.*d*, *nec3*.*e*, *nec3*.*l* and *nec3*.*m* mutants at seven days after pathogen inoculations compared to the mock controls treated with water. We observed that the Serotonin fed *nec3* mutants and wild-type plants displayed relatively reduced-sized disease lesions and show no observed chlorosis on the secondary leaf (S3A and S3B Fig). However, an opposite trend was observed in the tryptamine-fed plants, where the lesion size was relatively increased (S3A and S3B Fig).

### DAB staining and electron microscopy

**Fig 4:**
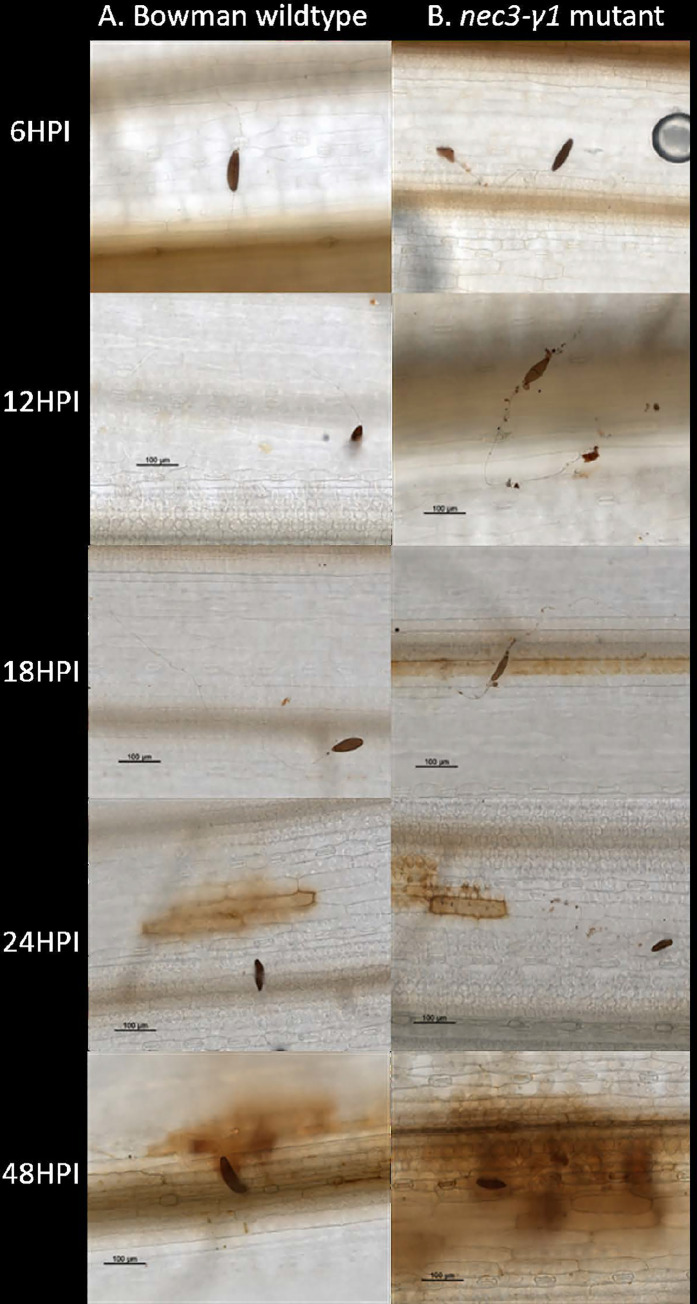
Microscopic visualization for comparison of the barley *nec3-γ1* mutant and Bowman wildtype for ROS production and pathogen growth on the leaf surface during infection process. On the left is the Bowman wildtype and on the right is the *nec3-γ1* mutant ROS production during infection of *Bipolaris sorokiniana* isolate ND85F at 6, 12, 18, 24, 48 hours post inoculation (HPI), where multiple branching of germ tubes, localized HR, and mutant specific interaction with the cuticle/of the barley leaves is observed as early as 12 hours post inoculation.

As the *nec3* lesions expand more rapidly and are larger than those of the WT genotypes it was expected that the mutants would express differential ROS production. DAB staining associated with most of the *B*. *sorokiniana* penetration sites was observed as early as 12 hpi on *nec3-γ1* inoculated leaves (Fig 4). The DAB staining associated with pathogen penetration sites was only observed in the resistant WT Bowman line after 18 hpi. During the later time-points (24–36 hpi) the DAB staining associated with *B*. *sorokiniana* penetration and colonization in the susceptible *nec3-γ1* mutant, rapidly increased to neighboring host epidermal cells, as well as underlying mesophyll cells. However, in the resistant WT Bowman seedlings, the DAB staining that started to appear at ~ 24 hpi had a higher intensity but remained limited to a few cells adjacent to the penetration site and did not expand at the later time-points during the infection process as was observed with the *nec3-γ1* mutant (Fig 4)

**Fig 5:**
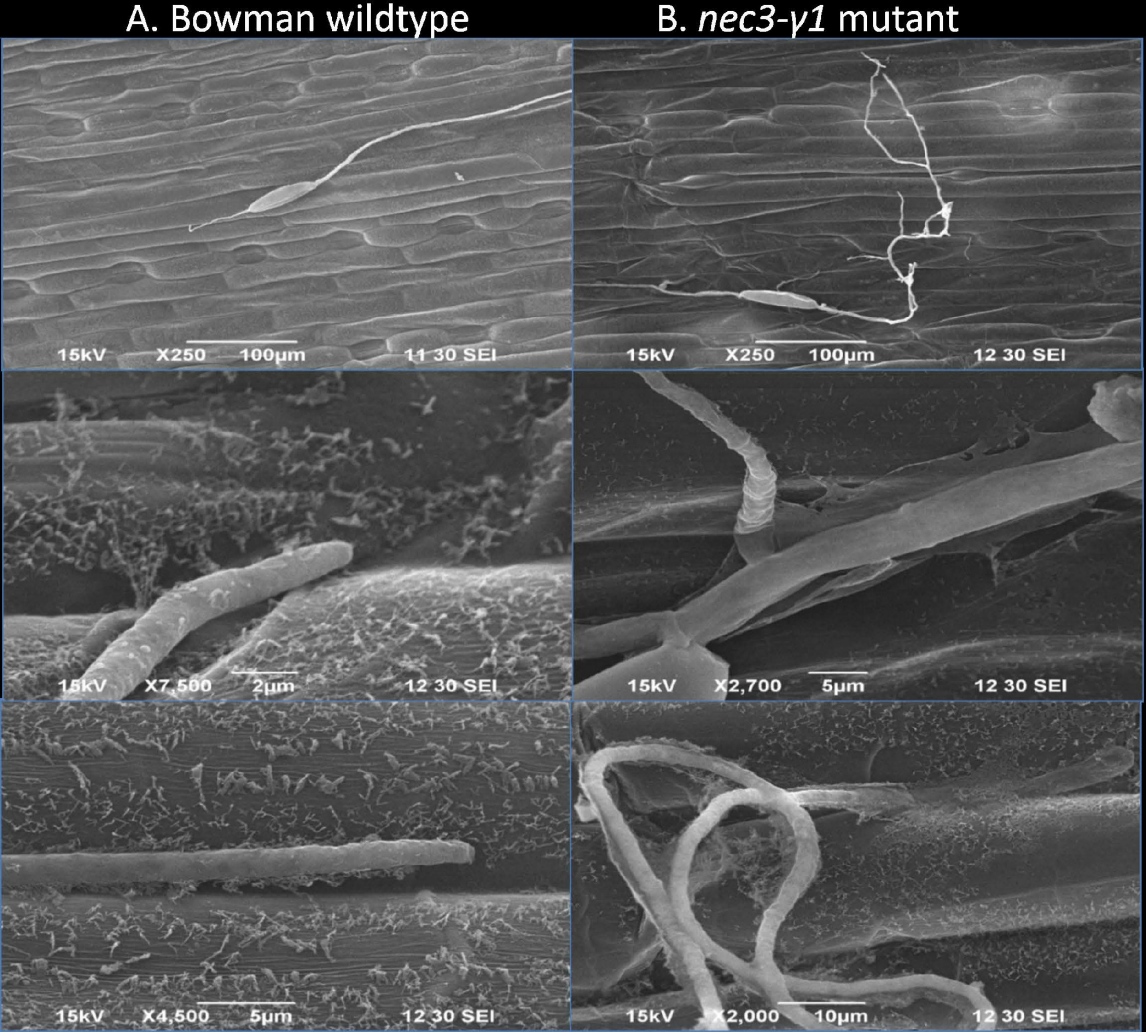
Electron Micrograph of the pathogen *Bipolaris sorokiniana* growth on the leaf surface of the barley *nec3-γ1* mutant and Bowman wildtype during the infection process. 6A. On the left is the Bowman wildtype with normal *B*. *sorokiniana* isolate ND85F growth and intact cutin layer of host at 12 hours post inoculation. 6B. On the right is abnormal growth of *B*. *sorokiniana* isolate ND85F with multiple branching of germ tubes and mutant specific interaction with the cuticle of the barley leaves at 12 hours post inoculation.

Time course light microscopy following DAB staining post *B*. *sorokiniana* isolate ND85F inoculations showed normal spore germination and germ tube growth on WT Bowman with germ tubes growing from each end of the conidium with little to no germ tube branching (Figs 4 and 5). Spore germination on the *nec3-γ1* mutant appeared normal, however, aberrant germ tube branching was consistently observed compared to the growth on WT Bowman (Figs 4 and 5). Also, after DAB staining of the *nec3-γ1* mutant, it was consistently observed that dark-colored debris would accumulate along the path of the germ tube growth across the leaf surface (Fig 4B) compared to the growth on WT Bowman (Fig 4A). As it could not be discerned if the cellular debris accumulating along the germ tube growth on the *nec3-γ1* mutant was host or pathogen-derived, electron microscopy was used to generate higher resolution images during the early infection process. These images showed that the debris was derived from the host cuticle peeling away from the leaf surface around the region of germ tube contact with the leaf surface (Fig 5).

### nec3 genetic map development

A genetic map was developed using homozygous F_2_ recombinant progeny from the cross between *nec3-γ1* and Quest (*γ1* x Q) to map the *nec3* gene. Homozygous mutant individuals were identified by inducing the *nec3* phenotype with *B*. *sorokiniana* isolate ND85F. Due to the typical recessive nature of mutant genes, a 3:1 wild type: mutant ratio was expected. Unfortunately, a lethal chlorophyll-/albino background mutation killed 54 of the 200 F_2_ progeny assayed. The albino mutation was not linked to *nec3* and was also segregated in a recessive 3:1 single gene manner (χ^2^ = 0.32). After accounting for the albino plants that died 33 of the 146 surviving plants developed the *nec3* phenotype fitting the expected 3:1 ratio (χ^2^ = 0.17), indicating that *nec3* was segregating as a single mutant gene in a recessive manner.

**Fig 6:**
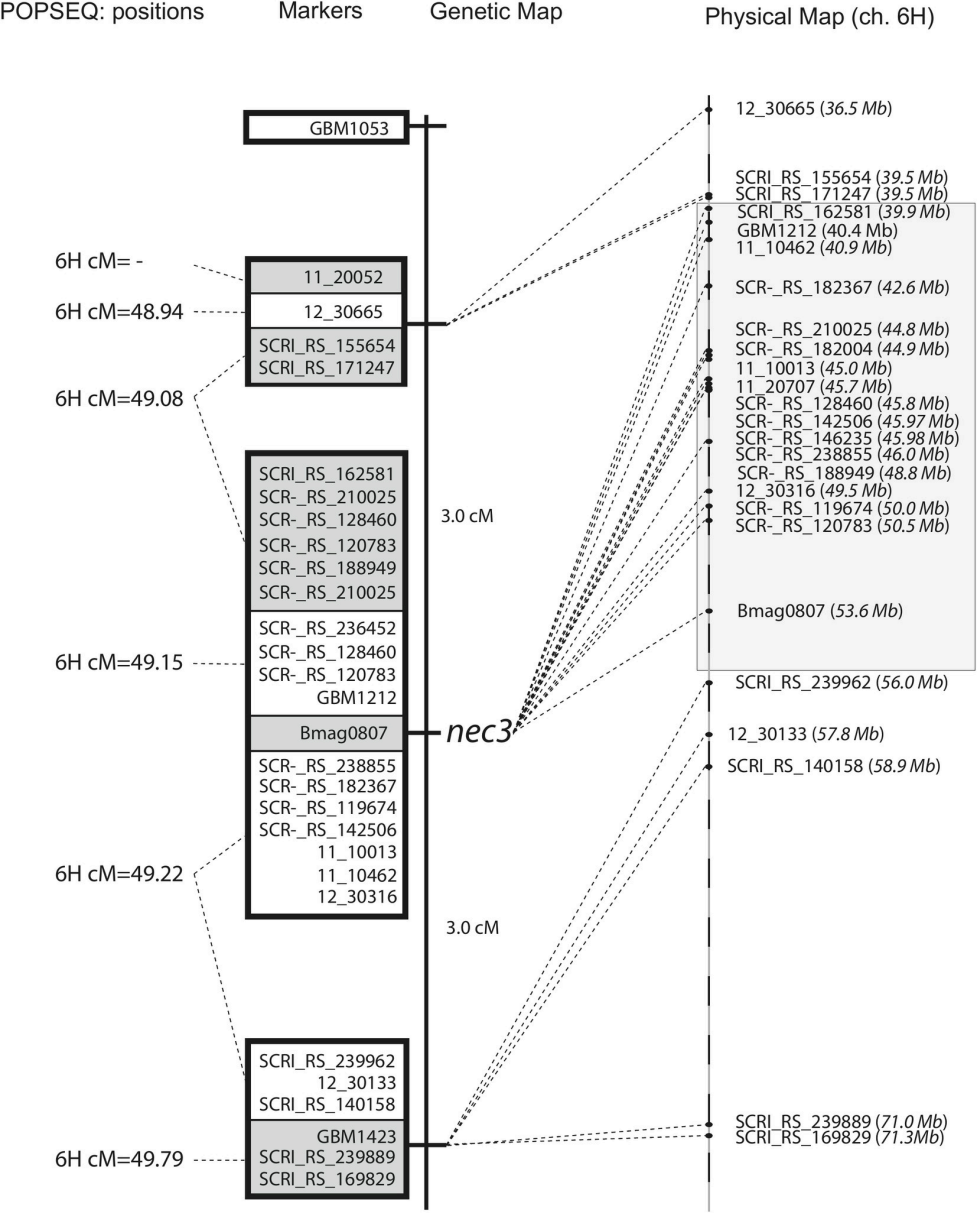
Genetic and physical map of the *nec3* region. On the left is the genetic map developed from 33 homozygous mutant F_2_ individuals (representing 66 recombinant gametes) from the cross between *nec3-γ1* and Quest. The genetic distances, based on recombination frequency is shown with the boxes indicating cosegregating markers based on the F_2_ map and white or gray shading indicating cosegregating markers based on POPSEQ consensus positions, which are given on the far left. The relative physical location of the markers is shown on the right, which were derived from the new released whole genome assembly from the IPK database.

To map the *Nec3* gene, the chromosome 6H region was saturated with molecular markers. According to the IPK cv Morex genomic sequences and POPSEQ positions [33,34], the *Nec3* gene co-segregated with marker GBM1212 located at 49.07cM on chromosome 6H. The proximal flanking marker GBM1423 was positioned at 49.22cM, 0.15cM proximal of GBM1212 and the distal flanking marker GBM1053 was positioned at 53.47cM, 4.4cM distal of GBM1212 (Fig 6) [34]. Thus, the *Nec3* gene was delimited to a relatively large genetic interval of 4.55 cM. To further delimit the *Nec3* region an additional 29 SNP markers were genetically anchored to the *nec3* region of barley chromosome 6H using the *γ1* x Q population. The positions of the 29 SNP markers on the genetic map were in perfect linear order with the newly released barley genome sequence and the barley POPSEQ positions. The physical *nec3* region was flanked by the SNP marker SCRI_RS_171247 distally at pseudomolecule position 39.5 Mb and marker SCRI_RS_239962 proximally at 56.0 Mb on chromosome 6H, delimiting the region to ~ 0.14 cM based on the barley POPSEQ positions correlating to a physical region spanning ~ 16.5 Mb containing 149 high confidence genes according to the newly release barley genome sequence and annotation (Fig 6). The exome capture was run on the mutants *nec3*.*l* and *nec3*.*m* along with the wildtype Steptoe using the capture array 120426_Barley_BEC_D04. The exome capture sequencing data were analyzed to identify potential deletions within the 149 high confidence candidate genes in the *nec3* region based on the WGA Morex sequence released in 2019 [34]. A total of 11 genes in the *nec3* region were not represented in the exome capture probe set, as shown in S3 Table. The 138 annotated high confidence genes (S2 Table) present in the exome probe set and captured in the *nec3* region were analyzed in WT Steptoe and the allelic mutants *nec3*.*l* and *nec3*.*m* (NCBI submission BioSample accessions SAMN22109510, SAMN22109511, SAMN22109512). No deletions were observed in the exons of the 138 genes from the three independent mutants.

### RNAseq and candidate gene identification

**Fig 7:**
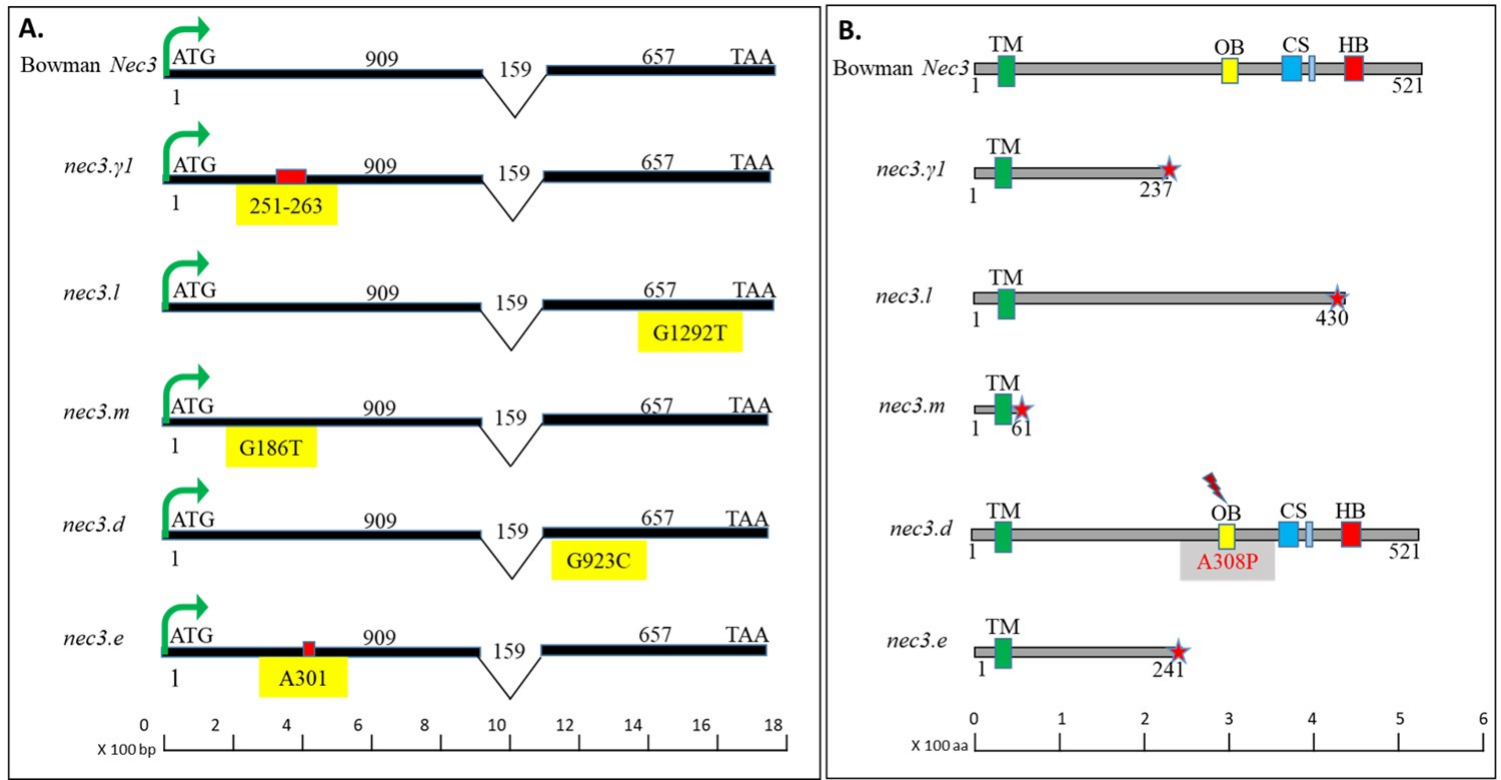
The allele analysis and protein polymorphism of the barley *nec3* mutants and Bowman wildtype. A. The genomic and cDNA structures for the barley *Nec3* gene in bowman wildtype and *nec3* mutants are shown from top to bottom (Bowman wildtype, *nec3-γ1*, *nec3*.l, *nec3*.m, *nec3*.d and *nec3*.e), where genomic and predicted mRNA structures are represented to scale with exon (black), intron (black Vs), start codon (ATG) and stop codon (TAA) and the mutations are denoted in the yellow box, where deletions are represented by red box above them. B. The barley *Nec3* protein structure in bowman wildtype and *nec3* mutants are shown from top to bottom (Bowman wildtype, *nec3-γ1*, *nec3*.l, *nec3*.m, *nec3*.d and *nec3*.e), where gray represent protein length, green bar represents transmembrane domain (TM), yellow bar represents oxygen binding (OB) and activation conserved residue AGxDTT, the blue bars represent the clade signatures (CS) with conserved residues ExxR and P(E)R(F) and red bars represent the heme binding (HB) with conserved residues as FxxGxRxCxG of the p450 clade. The star shows the truncated protein in the *nec3-γ1*, *nec3*.l, *nec3*.m, and *nec3*.e mutant and *nec3*.*d* has a A308P substitution represented by red lightening sign in the conserved residue of Oxygen binding domain of AGxDTT to PGxDTT, in the logos of the p450 protein family in plants.

RNAseq analysis was performed on the *nec3-γ1* mutant and WT Bowman post-inoculation with *B*. *sorokiniana* isolate ND85F to analyze the 11 genes in the delimited region missing from the exome capture analysis. The RNAseq also allowed for a thorough analysis of the global regulation of the transcriptomes in WT Bowman and the *nec3-γ1* mutant generated in the Bowman background upon *B*. *sorokiniana* isolate ND85F inoculation. To identify candidate genes, the total reads obtained and percentage alignment to the Morex reference genome was analyzed as well as comparative analysis between mutant and WT Bowman reads (S4 Table) (GenBank Bio project PRJNA666939). The RNAseq analysis at 72 hpi identified a total of 10,473 differentially expressed genes **(**DEGs) with greater than a threefold change (5,171 upregulated and 5,303 downregulated) in the *nec3-γ1* mutant compared to the non-inoculated *nec3-γ1* mutant (Accession SRR12763054-SRR12763062). In resistant WT Bowman, 5,463 DEGs (2,803 upregulated and 2,661 downregulated) were identified in comparison to the non-inoculated WT Bowman control. Interestingly, the comparison between Bowman and *nec3-γ1* mutant transcriptome profiles during *B*. *sorokiniana* isolate ND85F infection process revealed 3 genes upregulated in Bowman and downregulated in *nec3-γ1* mutant and nine genes were downregulated in Bowman and upregulated in *nec3-γ1* mutant (S4 Fig and S4 Table). The comparative analysis of the RNAseq data for the 11 genes missing from the exome capture probe set identified a 13 nucleotide deletion in the predicted exon1 of the cytochrome P450 gene HORVU.MOREX.r2.6HG0460850 in the *nec3-γ1* mutant (Fig 7). Eight of the eleven genes missing from the exome capture probe were not detected at control or in the pathogen-induced samples at the tested time-point in both WT Bowman or the *nec3-γ1* mutant (S3 Table), fold changes reported as non-significant (NS). Interestingly, the candidate *Nec3* gene HORVU.MOREX.r2.6HG0460850 was upregulated 1,126 fold in susceptible *nec3-γ1* mutant after pathogen inoculation and only upregulated 118 folds in WT Bowman after pathogen inoculation (S3 Table).

### Cytochrome P450 mutant allele analysis and functional characterization

The candidate *Nec3* Cytochrome P450 gene (HORVU.MOREX.r2.6HG0460850) was the only gene identified in the RNAseq analysis in the *nec3* region that contained a mutation in *nec3-γ1*. Analysis of the other four independent *nec3* mutants via PCR amplicon sequencing compared to their respective WT genotypes showed that all five *nec3* alleles contained a mutation that would disrupt the function of the predicted translated protein. The *Nec3* gene (HORVU.MOREX.r2.6HG0460850) encodes a 1,566 bp gene (supported by RNAseq data) predicted to be translated into a 521 aa protein with an N-terminal transmembrane domain and a C-terminal p450 domain. The p450 domain has three conserved motifs, the oxygen-binding (AGxDTT), clade signatures (ExxR) (P(E)R(F)) and heme-binding (FxxGxRxCxG) motifs (Fig 7) [35]. The *nec3-γ1* mutant has a 13-nucleotide deletion at position 251 to 263 bp in exon1, which is predicted to result in a premature stop codon and truncated protein of 237 aa, eliminating the entire p450 domain (Fig 7). The G186T nucleotide conversion in the *nec3*.*m* was predicted to produce a premature STOP codon and a truncated protein of 61 aa eliminating the p450 domain (Fig 7). The *nec3*.*e* mutant contained the single nucleotide deletion A301 that was predicted to cause a frameshift resulting in a premature STOP codon and predicted truncated protein of 241 aa also eliminating the p450 domain (Fig 7). The G1292T nucleotide conversion in the *nec3*.*l* mutant was predicted to produce a premature STOP codon at aa position 430, eliminating the heme-binding motif present in the p450 domain (Fig 7). Intriguingly, the allelic *nec3*.*d* mutant that produces an atypical *nec3* phenotype with the typical DLMM lesions that contain dark phenolics and a relatively smaller lesion size compared with the other *nec3* mutants had a G923C nucleotide substitution mutation, which resulted in the predicted A308P aa conversion. This aa substitution is present in the conserved residues of the oxygen-binding (AGxDTT) motif of the p450 domain, yet was still predicted to produce a full-length protein. Thus, it is possible that the *nec3*.*d* mutant has compromised function but maintains partial T5H function (Fig 7). Tryptamine to serotonin conversion in the *in vitro* reaction mediated by yeast expressed barley Nec3_Δ26_ protein could not be convincingly detected. LC-MS analysis identified an artifact peak close to the expected peak position for serotonin (S7 Fig). This Type I error was mitigated using multiple controls such as Tryptamine only and no Nec3 in the reaction, which resulted in a similar artifact peak with a minor shift in the acquisition time.

## Discussion

The cytochrome P450 gene (HORVU.MOREX.r2.6HG0460850) was identified as the *Nec3* (necrotic 3) gene. Barley *nec3* mutants that produce the atypical and distinct *nec3* phenotype, large cream to orange necrotic lesions lacking the dark pigmentation indicative of the accumulation of serotonin phenolic compounds typical of previously reported DLMMs, were characterized in the cv Bowman, Steptoe, Proctor and Villa backgrounds. Four of the independent mutants had deletions or nucleotide substitutions in the cytochrome P450 gene that resulted in predicted nonfunctional truncated proteins. Interestingly, a fifth independent mutant shown to be allelic to *nec3* produced the typical DLMM phenotype with smaller dark necrotic lesions containing serotonin or phenolics buildup (Fig 1). This *nec3* mutant with a single nucleotide substitution presents a unique opportunity to investigate the role that serotonin [32] or phenolics accumulation in PCD necrotic lesions play in the sequestration of lesion expansion or pathogen colonization during HR responses. It was also discovered that *nec3* is not a true DLMM as it is only expressed when the plants are challenged by diverse specialized barley pathogens.

Originally the *nec3* mutants were classified as LMMs [29], referred to as disease lesion mimic mutants (DLMMs) here, that spontaneously develop necrotic lesions when they reach a certain developmental stage. However, contrary to these observations and the DLMM designation, we determined that the *nec3* phenotype is only expressed when elicited by a diverse taxonomy of pathogens. The *nec3* phenotype is possibly triggered through PAMP elicitor recognition, chitin in the case of fungal pathogens, during pathogen challenge, entry, and host colonization. Previous research and observations that led to the description of *nec3* as a DLMM were conducted under less controlled greenhouse and field environments, where the *nec3* phenotype was consistently expressed without apparent biotic or abiotic stress induction [28]. However, in the same study, [28] it was observed that mutant seedlings grown in growth chambers for transcriptome analysis did not exhibit the *nec3* phenotype. In our screen of a variety Bowman mutant population for mutants of the dominant *B*. *sorokiniana* isolate ND90Pr susceptibility gene [36], the *nec3-γ1* mutant was identified. The *nec3-γ1* mutant exhibited the phenotype consistent with the previously described and characterized *nec3* mutants [36]. However, the phenotype appeared only after inoculation with the pathogen leading us to speculate that the *nec3* characteristic lesions were induced by pathogen challenge. To test the hypothesis that the *nec3* phenotype was only induced by pathogen infection, a sterile environmental condition experiment was conducted. Upon conducting experiments under sterile conditions with proper controls, it was determined that the *nec3* phenotype was induced in all the *nec3* mutants (*nec3-γ1*, *nec3*.*d*, *nec3*.*e nec3*.*l*, and *nec3*.*m*,) by the necrotrophic ascomycete fungal pathogens *B*. *sorokiniana*, *P*. *teres* f. *teres*, and *P*. *teres* f. *maculata*, as well as the biotrophic ascomycete *Blumeria graminis* (Fig 2). During host penetration, these fungal pathogens are known to form appressoria-like structures and penetration pegs that puncture the cell wall and plasma membrane causing cellular damage [37–39]. The host disruption and pathogen penetrative structures are also accompanied by proteinaceous and secondary metabolite effector molecules that induce host immune responses that induce temporally regulated and spatially confined succinct PCD responses. These disruptive infection processes that induce PTI/DTI signaling also result in the upregulation of PR proteins that arrest pathogen colonization and induce systemic acquired resistance (SAR) [40].

The bacterial pathogen *X*. *translucens*, which causes tissue damage by employing the penetrative Type-III secretion system to deliver virulence effectors also induced the *nec3* phenotype. During bacterial colonization increased extracellular polysaccharide (EPS) exudates are also released to facilitate communication between bacteria, which increases the chances of a successful infection. Bacterial pathogens also utilize enzymes and other molecules to increase the permeability of the cell wall. This leakiness allows nutrients to be acquired by the bacteria and facilitating its colonization. Collectively, the loss of cellular integrity induced by the bacterial pathogen may induce the *nec3* phenotype or a PAMP-like bacterial elicitor also triggers the *nec3* phenotype, however, our experiments determined that the bacterial PAMP flg22 didn’t induce the *nec3* phenotype.

Interestingly, the *nec3* phenotype was not elicited by either virulent or avirulent races of the basidiomycete biotrophic pathogen *P*. *graminis* f. sp. *tritici*, whose strategy is “incognito” host entry through the stomata. It is hypothesized that *P*. *graminis* f. sp. *tritici* hijacks the stomatal aperture regulation of the cereal hosts to enter through the natural openings at night without being detected [41,42]. The *nec3-γ1* mutant in Bowman background contains the stem rust resistance gene *Rpg1* [43,44], which elicits an effective race-specific immunity response yet did not elicit the *nec3* phenotype. Recent research has reported that *Rpg1*-mediated defenses are non-HR responses [41,45], thus, suggesting that the *nec3* phenotype may still be elicited by HR-mediated resistance responses.

Necrotrophic fungal pathogens produce proteinaceous, non-proteinaceous, and secondary metabolite effectors that initiate host PCD to colonize the dead and dying tissue via necrotrophic effector-triggered susceptibility (NETS) [12,13,46,47]. To determine the nature of the elicitor/s of the *nec3* response a pathogen culture filtrate infiltration assay was performed. *B*. *sorokiniana* crude culture filtrates elicited the distinct *nec3* PCD phenotype on the mutants and characteristic dark pigmented lesion in the susceptible barley line Steptoe, but not in the resistant cv Bowman (S1 Fig). However, in the *nec3*.*d* mutant, culture filtrates didn’t produce the pronounced dark pigmented lesions as seen with the *B*. *sorokiniana* isolate ND85F inoculations on *nec3*.*d*. Pronase treatment of the culture-filtrate did not abolish the elicitation of the distinct *nec3* lesions at the infiltration site. Thus, we speculate that the pathogen effector/s in the culture filtrates that elicit the *nec3* phenotype is/are not proteinaceous effector/s but rather represent a PAMP such as chitin, a secondary metabolite, a non-proteinaceous toxin, an RNA molecule or a tightly folded small protein. However, it cannot be ruled out that *nec3* is involved in suppressing PCD responses elicited by DAMPs which include cell wall components, eATP, eDNA, or other endogenous molecules disrupted during the infection process as recognition of disrupted self is seen across all multicellular life, including algae, fungi, fish, insects, mammals and plants [48]. However, our experiments showed that OGs did not elicit the *nec3* phenotype.

To identify the *nec3* gene a genetic map was generated with F_2_ recombinant progeny from the cross between *nec3-γ1* (Bowman background) and the barley variety Quest (referred to as the *γ1* x Q population). The genetic mapping delimited the *nec3* region to ~0.14 cM on barley chromosome 6H between the flanking markers SCRI_RS_155654 and SCRI_RS_239962. The SNP markers SCRI_RS_155654 resides between 39,543,736–39,543,856 base pair (bp) and SCRI_RS_239962 is situated between 56,035,754–56,035,852 bp delimiting *Nec3* to a physical distance of ~16.49 Mb on barley chromosome 6H containing 149 high confidence annotated genes. We further analyzed these genes by carrying out whole genome exome capture sequencing on WT Bowman and WT Steptoe and three of the independent *nec3* mutants, *nec3-γ1*, *nec3-l* and *nec3-m*. However, the exome capture probe library only contained probes for 138 of the 149 annotated HC genes present in the genetically delimited *nec3* region based on the recently refined assembly and annotation of the barley Morex reference genome (2019) [34]. Therefore, it is important to correlate the available probe set with the latest whole-genome assembly to include the potentially unannotated genes in the analysis to find the causal gene of a trait for any species. The absence of any mutated genes by exome capture analysis within the *nec3* region and the identification of the 11 annotated HC genes in the *nec3* region that are missing from the exome capture probe set led to the comparative analysis approach of RNA sequencing of Bowman WT and the *nec3*-*γ1* mutant control and induced by *B*. *sorokiniana* infection (S4 Table). Utilizing the RNAseq data for comparative analysis a 13 bp deletion was identified in the HORVU.MOREX.r2.6HG0460850 HC gene model in the *nec3- γ1* mutant. Utilizing PCR amplification and Sanger sequencing we further confirmed mutations that would result in predicted nonfunctional truncated proteins in all the four independent mutants that produce the typical *nec3* phenotype (*nec3-γ1*, *nec3*.e, *nec3*.*l*, and *nec3*.*m*) and a critical amino acid substitution in the *nec3*.*d* allelic mutant that expresses the atypical dark necrotic spots (Figs 1 and 7).

The presence of irradiation-induced deletions and nucleic acid substitutions in five independent mutants confirmed that HORVU.MOREX.r2.6HG0460850 is the functional *Nec3* gene. The HORVU.MOREX.r2.6HG0460850 HC gene model is predicted to encode a 521 amino acid Cytochrome P450 family protein. Cytochrome P450s are heme-thiolate proteins and represent one of the largest superfamilies of proteins with enzymatic activity. Within the superfamily, the amino acid sequence conservation is very low and only three residues belonging to the conserved sequence motif (CSM) are absolutely conserved. However, the general topography and structural folding are highly conserved across the members [49]. Only the CYP51 gene family of P450s is conserved among plant, fungi and animal phyla with the presence of CYP51 orthologs in the bacterium *Mycobacterium tuberculosis*, possibly due to lateral gene transfer [35].

Sequence comparison and phylogenetic analyses of the NEC3 protein places it in the CYP71 clan as a cytochrome P450 71A1-like protein (http://www.p450.kvl.dk/blast.html), suggesting involvement in plant lineage-specific metabolism [50] (S5 Fig). Upon annotating the putative protein-coding sequences of the five allelic *nec3* mutants, four were predicted to encode premature stop codons due to the induced mutations and one had a single nucleotide substitution that resulted in an A308P amino acid conversion in the predicted oxygen binding and activation domain in the conserved P450 residues (Fig 7). The data showed that upon pathogen infection, this mutant *nec3*.*d* allele was induced and predicted to be translated into a full-length protein with a nonfunctional oxygen-binding domain due to the single amino acid substitution (Fig 7). Interestingly, the *nec3*.*d* mutant although allelic to the truncated nonfunctional *nec3* alleles expresses comparatively smaller dark pigmented lesions presumed to be due to higher serotonin/phenolics accumulation. We hypothesize that the smaller lesion size of *nec3*.*d* compared to the typical cream-colored *nec3* phenotypes expressed by *nec3-γ1*, *nec3*.e, *nec3*.*l*, and *nec3*.*m* mutants were due to a partially functional Nec3 protein yet elicited the DLMM phenotype due to its compromised oxygen-binding domain. Another possibility to describe this anomaly is pathogen sequestration due to the serotonin/phenolics accumulation in the lesions. We assessed the role of exogenous root feeding of serotonin and tryptamine in these mutants and found that artificial availability of serotonin to supplement the deficiency did reduce the size of the *nec3* lesion and over-accumulation of tryptamine increased the lesion size in wildtype and mutants. Suggesting, that the sequential conversion of tryptamine into serotonin is mediated by Nec3, which is predicted to encode a tryptamine 5-hydroxylase (T5H) and exogenous application of upstream or downstream substrates in the pathway can modulate the final phenotypic outcome.

These observations and apparent phenotypic differences suggest that the oxygen binding domain plays a critical role in the NEC3 protein function in regulating PCD as well as the pathway leading to serotonin accumulation [32]. The *nec3*.*d* allele presents a unique tool for further functional characterization of the role of serotonin/phenolic deposition in necrotic lesions and their role in pathogen sequestration or the regulation of lesion expansion. As the question of how or why serotonin/phenolics build up in PCD induced necrotic lesions is an important question that still needs to be answered.

Interestingly, the Sekiguchi lesion (*sl*) mutant in rice produces orange tan necrotic lesions, similar to the barley *nec3* phenotype. The *sl* gene was identified via map-based cloning and shown to encode CYP71P1 in the cytochrome P450 monooxygenase family that shares 90.1% amino acid similarity and 86.7% identity with the barley *Nec3* protein (S6 Fig) [32]. The *sl* gene was shown to encode a Tryptamine 5-Hydroxylase (T5H) enzyme that converts tryptamine to serotonin in plants in the shikimic pathway [32]. The rice *sl* gene mutants were susceptible to the rice blast fungus (*Magnaporthe grisea*) and rice brown spot fungus (*Bipolaris oryzae*) and the *sl* necrotic lesions were also induced by the N-acetylchitooligosaccharide (chitin) elicitor [31]. The *sl* mutant susceptibility to rice blast was eliminated by exogenous application of serotonin in rice leaves [31]. The exogenously applied serotonin was deposited into the cell wall of the *sl* lesion tissue and these lesions with additional serotonin phenolics had restored dark pigmentation and restored resistance to the necrotrophic pathogen *B*. *oryzae* [32]. Thus, the *nec3*.*d* mutant containing the partially functional full-length protein may be able to convert Tryptamine to Serotonin but due to the critical A308P substitution has a leaky control on PCD and is not able to keep it in check upon pathogen recognition. We tested the T5H functional capability of Nec3 protein, however, we couldn’t convincingly detect the Tryptamine to serotonin conversion in the *in vitro* reaction mediated by yeast expressed barley Nec3_Δ26_ protein. Increased level of Serotonin due to root-feeding was able to reverse the *nec3* phenotype *in vivo*, nonetheless, failure to determine the in vitro T5H activity of Nec3 could possibly be attributed to its specificity to the species-specific reductase or requirement of additional *in vivo* activity components that are yet to be determined.

Plants have evolved oxidative polymerization of serotonin in the modification of cell walls as part of the physical barrier and phenolics deposition at the infection site during pathogen infection to inhibit pathogen growth and sequester them in the foci of dead cells. This mechanism may also act as a signaling mechanism to sequester lesion expansion to preserve the leaf’s photosynthetic capability after reacting to pathogen challenge. The barley *nec3* mutants fail to regulate the PCD response, providing expanding necrotic lesions with no defined border. This was further exemplified by the *nec3*.*d* mutant that still accumulates phenolics and has a defined lesion border that sequesters the expansion of the lesion in the presence of the pathogen. This phenomenon is also observed in resistant WT Bowman. The RNAseq analysis showed that the barley *NEC3* gene is highly upregulated in WT Bowman and the *nec3- γ1* mutant, 72 hours post *B*. *sorokiniana* inoculation, thus the upregulation of the *sl* ortholog suggests an increased need to convert Tryptamine to Serotonin in barley as well because of its importance in regulating PCD-mediated immunity.

Necrotrophic fungal pathogens produce proteinaceous, non-proteinaceous, and secondary metabolite effectors that initiate host PCD to colonize the dead and dying tissue via necrotrophic effector-triggered susceptibility. To determine the nature of the elicitor/s of the *nec3* response a pathogen culture filtrate infiltration assay was performed. *B*. *sorokiniana* crude culture filtrates elicited the distinct *nec3* PCD phenotype on the mutants and characteristic dark pigmented lesion in the susceptible barley line Steptoe, but not in the resistant cv Bowman. However, in the *nec3*.*d* mutant, culture filtrates did not produce pronounced dark pigmented lesions as seen with the *B*. *sorokiniana* ND85F isolate inoculation on *nec3*.*d*. This suggests that this single amino acid substitution allows for the induction of enhanced PCD elicitation by the pathogen in the WT Bowman background yet possibly still retains partial function allowing for serotonin metabolism and build up in the lesions that still functions to sequester the pathogen and necrotic lesion expansion. The culture filtrate infiltration of the *nec3*.*d* mutant was indistinguishable from the other *nec3* mutants with no dark pigmentation in the absence of the pathogen. This suggested that post-elicitation, the *nec3*.*d* mutant behaves similarly to the other non-functional mutants but in the presence of the pathogen, it is still able to mount some level of serotonin biosynthesis and suppression of lesion expansion and pathogen growth.

The pronase treatment of the culture-filtrate did not abolish the elicitation of the distinct *nec3* lesions at the infiltration site. Thus, we can speculate that the pathogen effector/s in the *B*. *sorokiniana* culture filtrates that elicit the *nec3* phenotype is not a proteinaceous effector but similar to the *sl* mutant in rice may be elicited by a PAMP, possibly chitin as it elicited the *sl* phenotype and possibly elicits *nec3* in barley as well. This would suggest that the *nec3* necrosis may be elicited by chitin through extracellular LysM domains, present on barley orthologs of the rice LysM RLK, OsCERK1, and the LysM RLP CEBiP [51]. However, due to the observation that the *nec3* phenotype is also elicited by the bacterial pathogen *X*. *translucens* there is a possibility multiple effectors inducing PCD responses suppressed by *Nec3*.

In Arabidopsis, the CYP84A1 EMS mutant was shown to have altered lignin composition [52]. Thus, alterations of the cell wall composition could affect the pathogen’s spatiotemporal interactions with the host at the leaf surface, which we tested microscopically in the *nec3-γ1* mutant. Upon microscopic observations of *B*. *sorokiniana* growth pattern on the *nec3* mutant compared to WT Bowman it was observed that the pathogen’s infection hyphae interaction with the host cuticle was abnormal showing that the cuticle was unstable and peeled away from the *nec3-γ1* leaf surface, where there was contact (Fig 5B). This aberrant interaction apparently disrupted the signaling in the pathogen’s infection hyphae. When growing across the *nec3* mutant leaf surface the *B*. *sorokiniana* hyphae branched profusely and produced surface debris apparently due to unstable cuticle along the germ tubes (Fig 5). The profuse branching of pathogen and plant cell surface destabilization could result in many host-pathogen contact points with the *nec3* mutants allowing the plants to recognize more PAMP molecules, possibly chitin fragments. This aberrant interaction possibly leads to the rapid ROS production in *nec3 cyp71A1* mutants and the runaway cell death due to the lack of serotonin accumulation, which may play a role in the sequestration of PCD mediated lesion expansion of the necrotic lesions similar to the *sl* mutant in rice. The DAB assays supported this conclusion as the *nec3-γ1* mutant produced ROS when infected by the necrotrophic pathogen *B*. *sorokiniana* as early as 12 hpi as compared to a delayed ROS in the susceptible Steptoe and resistant Bowman, which was detected at 18 and 24 hpi, respectively (Fig 4).

Here we report on the identification of the barley *Nec3* gene as a P450 CYP71A1 cytochrome oxidase that plays a role in suppressing PCD responses that are initiated by diverse adapted barley pathogens. We hypothesize that *Nec3* is a negative regulator of the PCD and is an ortholog of the *sl* gene identified and characterized in rice, which plays a role in serotonin biosynthesis and buildup in necrotic lesions. The barley *Nec3* gene provides a valuable resource to study the control of PCD and HR responses and our collection of mutant alleles especially the *nec3*.*d* mutant will be an excellent tool for determining the role of serotonin and possibly phenolic compound deposition in necrotic lesions for the sequestration of pathogen colonization or lesion expansion.

## Materials and methods

### Elicitation of nec3 phenotype

An isolation box experiment was performed on the wildtype (WT) cv Bowman and *nec3-γ1* (Bowman background) seeds over a month to observe the *nec3* phenotype independent of pathogen challenge with details in S1 Appendix.

### Pathogens that induce nec3 phenotypes

To determine if diverse pathogens could induce the *nec3* phenotype, inoculations of the mutant line *nec3-γ1* and WT lines were performed using diverse fungal pathogens, including *B*. *sorokiniana*, *Pyrenophora teres* f. *teres*, *Pyrenophora teres* f. *maculata*, *Pyrenophora tritici-repentis*, *Parastagonospora nodorum*, *Cercospora beticola*, and virulent and avirulent races of *Puccinia graminis* f. sp. *tritici*. The barley bacterial pathogen *Xanthomonas translucens* pv *undulosa* was also used to inoculate the plants. The materials and methods followed are described in S1 Appendix.

### Infiltrations with DAMPs

Culture filtrate infiltrations were performed for *B*. *sorokiniana* isolate ND85F 1mL of the suspension (10-20k spores) was taken from the plates and added to 75-100mL of Fries media (56). The flasks containing Fries media and *B*. *sorokiniana* spores were incubated at 26°C in the dark for three days with shaking at 100rpm then placed in the dark at room temperature with continued shaking for an additional four days. Following 7 days of growth, the exudates were filtered with Miracloth and concentrated using a 15mL Microsep Advance Centrifugal Device with a 3kD size exclusion to concentrate the exudates ~8x. A syringe without a needle was used to infiltrate four secondary leaves of WT Bowman, WT Steptoe, *nec3-γ1*, WT Quest, *nec3*.*l* and *nec3*.*m*, *nec3*.d and *nec3*.e. The four treatments consisted of; 1) concentrated exudates + Fries Media, 2) concentrated exudates + MOPs buffer, 3) concentrated exudates + MOPs + Pronase (Sigma), and 4) Fries media + MOPS + Pronase. All treatments, concentrated exudates + Fries media, and concentrated exudates + MOPS were at 1:1 ratio with specifications performed according to Liu 2004 (57). Pronase treatments were performed at 1mg/mL and leaves were scored 4 and 7 days after infiltrations.

Infiltrations were also performed with trigalacturonic acid, Flg22 and Chitin. Wildtype Steptoe and Bowman, *nec3*.*l* and *nec3-γ1* were infiltrated with the DAMP trigalacturonic acid at 10mg/mL, 1mg/mL and 0.1mg/mL. with Flg22, a PAMP at 1mg/mL and Chitin at 2μg/ml. All infiltrations with the controls were performed at the two-leaf stage. Following infiltration, plants were kept in a growth chamber with a 14-hour photoperiod at 22°C and 10 hours of dark at 18°C. Plants were observed every day up to seven days after infiltration.

### DAB staining

To observe ROS at the site of infection, five 3 cm leaf samples were collected from secondary leaves at 6, 12, 18, 24 and 48 hpi from WT Bowman and *nec3-γ1* mutant seedlings inoculated with *B*. *sorokiniana* isolate ND85F. After detachment, the leaves were immediately transferred to 10 ml of freshly prepared 1mg/ml DAB (Sigma Aldrich, MO) solution (pH 3.6) in 15 ml tubes following the protocol described by [53].

### Electron microscopy

Electron microscopy was performed on leaves collected from WT Bowman and *nec3-γ1* mutant seedlings at 12 hpi with *B*. *sorokiniana* isolate ND85F. The leaves were collected and cut into squares with a razor blade, fixed in 2.5% glutaraldehyde in sodium phosphate buffer (Tousimis, Rockville, Maryland USA) and stored at 4°C overnight. The sectioned leaf samples were rinsed with distilled water followed by rinsing with sodium phosphate buffer 1M solution at 7.4 pH and then dehydrated using eight washes of a graded alcohol series from 30% to 100% ethanol with incremental concentrations increased by 10% for each wash. The leaf samples were critical-point dried using an Autosamdri-810 critical point drier (Tousimis, Rockville, Maryland USA) with liquid carbon dioxide as the transitional fluid. The dried leaf samples were attached to aluminum mounts with silver paint (SPI Supplies, West Chester, Pennsylvania USA) and sputter-coated with gold (Cressington sputter coater Redding, California USA). Images were obtained using a JEOL JSM-6490LV scanning electron microscope operating at an accelerating voltage of 15 kV.

### Allelism crosses

The *nec3-γ1* mutant identified in this study was crossed with *nec3*.*1*, *nec3*.*m*, *nec3*.*d* and *nec3*.*e*. The resulting F_1_ seed was planted in the field with WT parental lines Steptoe and Bowman and phenotyped from seedling to adult plant stages. The F_2_ progeny were planted in the greenhouse and inoculated with the *B*. *sorokiniana* isolate ND85F and the nec3 phenotype/disease was rated on the secondary leaves of the seedlings. The phenotyping was performed in the field and greenhouse to take advantage of the entire year. This was made possible due to the consistency of elicitation of the *nec3* phenotype when grown adjacent to susceptible spreader rows inoculated with the *B*. *sorokiniana* isolate ND85F in the field and when inoculated in the greenhouse with *B*. *sorokiniana* isolate ND85F.

### Nec3 map development

The original *nec3-γ1* M_2_ plant generated in the cv Bowman background was utilized as the female parent in a cross with the six-rowed cv Quest that was originally developed for malting and released by the University of Minnesota (59). Two hundred *nec3-γ1* x Quest F_2_ individuals were screened in the greenhouse for the *nec3* phenotype after inoculation with *B*. *sorokiniana* isolate ND85F as previously described and a chi-square test was used to determine the goodness of fit. Allelism tests had determined that *nec3-γ1* was a *nec3* mutant. Thus, to map the gene more precisely simple sequence repeat (SSR) and single nucleotide polymorphism (SNP) markers spanning the previously delimited *nec3* region near the centromere of barley chromosome 6HS were used to genotype the F_2_ individuals showing the homozygous *nec3* mutant phenotype. Unfortunately, a lethal chlorophyll/albino background mutation killed 54 of the 200 F_2_ progeny assayed. This background chlorophyll/albino mutation segregated in a recessive 3:1 single gene manner (χ^2^ = 0.32). This mutation was not linked to *nec3* and we accounted for the plants that died and the number of remaining plants that developed the *nec3* phenotype to calculate the genetic ratio. Genomic DNA was extracted from *nec3-γ1*, Quest and 33 homozygous *nec3-γ1* F_2_ progeny representing 66 recombinant gametes. Four microsatellite markers designated Bmag0807, GBM1053, GBM1212, and GBM1423 were PCR amplified using oligonucleotide primers designed from the publicly available probe sequences mined from GrainGenes [54].

### PCR-GBS library preparation, Ion Torrent sequencing and SNP calling

A PCR genotyping-by-sequencing (PCR-GBS) panel was developed using SNP source file sequences mined from the T3 database [55] as previously described [56]. The POPSEQ positions were utilized from the IPK Barley BLAST Server [33] to identify 43 SNP markers that mapped to the *nec3* locus between the SSR markers GBM1053 and GBM1423. The parental lines *nec3-γ1* (cv Bowman) and cv Quest plus the 33 F_2_ homozygous *nec3* mutant recombinants were assayed. The PCR-GBS library preparation, Ion Torrent sequencing and SNP calling were performed as described before [56,57]. However, due to the low number of lines and markers, the library was sequenced on an Ion Torrent PGM 314 chip. The genetic map in the delimited region was generated manually and the POPSEQ positions of each marker were mined from the IPK genome browser.

### Physical map development and candidate gene identification

The 43 SNP markers were anchored to the IPK barley BLAST server (https://webblast.ipk-gatersleben.de/barley_ibsc/viroblast.php) based sequence and a physical position search was conducted against the 2019 Whole genome assembly of barley cv Morex sequences to create a minimum tiling path (MTP) physical map. The flanking markers GBM1053 and GBM1423 and the 43 SNP marker data on the *nec3-γ1* (cv Bowman) and cv Quest and 33 F_2_ recombinants (66 recombinant gametes) were used to develop the genetic map and were anchored to the WGS of barley to determine the physical map for the delimited *Nec3* region. The delimited *Nec3* region with flanking markers was used to list all the high confidence annotated genes in the region that were considered as candidate genes based on the 2019 Whole genome assembly of barley cv Morex [34].

### Exome capture and analysis

DNA was extracted from excised embryos of five germinated seeds of WT Bowman, WT Steptoe, *nec3- γ1* (Bowman background), *nec3*.*l* (Steptoe background), and *nec3*.*m* (Steptoe background) mutants. DNA extractions were performed on mechanically lysed samples using the PowerPlant Pro DNA isolation kit (Qiagen, CA), following the protocol described by Solanki et al. [57].

### RNAseq

The WT Bowman and *nec3*- *γ1* seedlings were grown for ~14 days until the secondary leaves were fully expanded in a growth chamber set at 14 hours light at 22°C and 10 hours dark at 19°C. The seedlings were inoculated with *B*. *sorokiniana* isolate ND85F following the procedure described earlier and the control seedlings were inoculated with water mixed with two drops of Tween20. Secondary leaf tissue was collected from three biological replicates (one individual seedling was considered as a biological replicate) from non-inoculated and inoculated seedlings at 72 hours post-inoculation (hpi) and total RNA was extracted using the RNeasy Mini kit (Qiagen, CA). The RNAseq library was constructed using TruSeq RNA library prep kit v2 (Illumina, CA) and sequenced on the Illumina NextSeq 500 (S1 Appendix). The final list was then produced by using the candidate genes in the mapped *Nec3* physical region with differential expression between the WT Bowman and *nec3-γ1* mutant DEGs during pathogen interaction. The reads pile-up from the differentially expressed candidate genes were aligned from WT Bowman and *nec3-γ1* mutant (Bowman background) and deletions were observed in the gene-specific reads pile-up on the CLC Genome Workbench 8.0.3.

### *Candidate nec3 gene allele analysis* and protein function characterization

The candidate *nec3* gene *HvCYP71-A1* was amplified from the *nec3-γ1* (Bowman background), WT Bowman, *nec3*.*1* (Steptoe background), *nec3*.*m* (Steptoe background), WT Steptoe, *nec3*.*d* (Proctor background), WT Proctor, *nec3*.*e* (Villa background) and WT Villa using the primer pairs Nec3_p450_F1 and Nec3_p450_R1; Nec3_p450_F3 and Nec3_p450_R2; Nec3_p450_F4 and Nec3_p450_R4 that were designed to produce overlapping amplicons with the respective fragment sizes of 1075, 563 and 404 bp (S4 Table). The purified PCR amplicons were sequenced using Sanger sequencing (Genscript, NJ). The overlapping PCR amplicon sequences were aligned and the specific mutations for each *nec3* mutant as compared to their respective wild type were determined.

The Nec3 proteins’ possible T5H activity to convert Tryptamine to Serotonin was tested *in vitro* as described in Fujiwara et al. (2010). The partial Nec3 protein (Nec3_Δ26_) was expressed in the X33 strain of *Pichia pastoris* with a c-terminal 6x His tag. The initial 26 amino acid encode a predicted transmembrane domain, thus was removed for the secreted protein expression. Secreted protein was purified by His-NTA column and analyzed by SDS-PAGE and western blot assays (S7 Fig). The 150 μl *in vitro* reaction consisted of 20 mM potassium phosphate (pH 7.25), 50 pmol/ml recombinant Nec3 protein, NADPH reductase from rabbit liver, and 100 μM tryptamine. Reactions were initiated by adding 1mm NADPH and incubated at 30°C for 30 min. Three controls, i.e., tryptamine (1 ng/μl) only, full reactions without Nec3, and without NADPH was carried out in parallel. After incubation samples were spiked with deuterated Serotonin as an internal control, diluted 1:5 with ice-cold methanol and centrifuged at maximum speed for 15 minutes to get the supernatant, which was concentrated overnight in the speed vacuum. Final samples were subjected to LC-MS analysis on Agilent 6495 triple quadrupole system for Tryptophan to Serotonin conversion analysis.

### Exogenous supplementation of Serotonin and Tryptamine

In three sets of treatments six replicates each of *nec3* mutants and their respective wildtypes were fed serotonin 150Μm, Tryptamine 5mM and water at 40 ml/plant through roots starting at 5 days old seedlings for every alternate day for total of 10 feedings. The plants were inoculated with *B*. *sorokiniana* isolate ND85F spores at 2000/ml on the 14 days old seedling by following previously described protocol and phenotyped on day 21^st^ after 7 days of disease infection.

## Supporting information

S1 Appendix Materials and methods. (DOCX)

S1 Table List of markers used to develop the barley *nec3* genetic and physical map on the chromosome 6H. The markers name, their sequence and physical position based on the 2019 Whole Genome Assembly of barley cultivar Morex.
(XLSX)

S2 Table The list of high confidence candidate genes in the delimited *nec3* region of the barley genome. The gene name, physical position, their presence on the Exome Capture Probe and annotation is based on the 2019 Whole Genome Assembly of barley cultivar Morex.
(XLSX)

S3 Table The list of high confidence candidate genes in the delimited *nec3* region of the barley genome that were not captured in the exome capture experiment were further analyzed for their expression during the spot blotch infection at 72 hours post inoculation. The gene name, and annotation is based on the 2019 Whole Genome Assembly of barley cultivar Morex and the relative normalized expression is reported in the Bowman wild type and *nec3-γ1* transcriptome profile of spot blotch infection, where no-significant change from control is denoted by the NS.
(XLSX)

S4 Table The List of primers used in the *Nec3* study. (XLSX)

S1 Fig Typical reactions of *Bipolaris sorokiniana* isolate ND85F culture filtrates on nec3 mutants and wildtype (wt) parental lines. Infiltrations of secondary leaves of barley lines Bowman wt, *nec3-γ1*, *nec3*.d, *nec3*.e., Steptoe wt, *nec3*.*l*, and *nec3*.*m* (left to right) with *Bipolaris sorokiniana* isolate ND85F culture filtrates (CF). The treatments from top to bottom (indicated to the left) were culture filtrates (CF) with Fries media (FM); CF with MOPS buffer (M); CF with M and pronase (P); and the control containing FM, M and P. Infiltrations were performed at the two-leaf stage and documented 4 and 7 days after infiltration. Pictures shown were taken at 7 days post infiltration.
(TIF)

S2 Fig The *nec3* phenotype was not induced by chitin infiltrations on the nec3 mutants and their respective wildtype barley plants. (A.) The panel shows the reaction to the chitin 2μg/ml infiltrations on the nec3 mutants from the left *nec3*.*d*, *nec3*.*γ1*, *nec3*.*e*, *nec3*.*l* and *nec3*.*m*, followed by the Bowman, Steptoe, Proctor, Villa wildtypes. (B.) The panel shows the reaction to the control buffer infiltrations on the nec3 mutants from the left *nec3*.*d*, *nec3*.*γ1*, *nec3*.*e*, *nec3*.*l* and *nec3*.*m*, followed by the Bowman, Steptoe, Proctor, Villa wildtypes.
(TIF)

S3 Fig The *nec3* phenotype induced by *Bipolaris sorokiniana* inoculation on the secondary leaf of the *nec3* mutants (A.) and their respective wildtype barley plants (B.), where plants were supplied with 40 ml/pot of water; serotonin 150 μg/ml and Tryptamine 5mM on every alternate days starting from 5 days old seedling for a total of 10 root feedings.
(TIF)

S4 Fig The transcriptome profile of the Bowman wildtype and nec3-γ1 mutant after 72 hours post inoculation of *Bipolaris sorokiniana* isolate ND85F. The venn-diagram represents the number of unique genes in a given class, where the blue and green oval represents Bowman downregulated and upregulated genes, respectively and the red and yellow oval represents nec3-γ1 mutant upregulated and downregulated genes, respectively (Top image). The bar graph shows the number of total genes present in each class Bowman and nec3-γ1 mutant up and down-regulated genes (Bottom image).
(TIF)

S5 Fig The NCBI blast phylogenetic tree of the barley Nec3 protein, where the branch represents the relationship between the close orthologs of the proteins in monocots. (TIF)

S6 Fig The similarity between the barley *Nec3* and the rice *sl* protein, where the dots represent same and the letters represents the different amino acid differences in the two proteins. (TIF)

S7 Fig LC-MS analysis of the Purified Nec3Δ26 protein’s possible Tryptamine 5-Hydroxylase activity to convert Tryptamine to Serotonin was tested in vitro as described in Fujiwara et al. (2010), using three controls, i.e., tryptamine (1 ng/μl) only, full reactions without Nec3Δ26, and without NADPH was carried out in parallel on Agilent 6495 triple quadrupole system. (TIF)
