# Supplementary material for: Mutations in a barley cytochrome P450 gene enhances pathogen induced programmed cell death and cutin layer instability
Source: PLoS Genet. 2021 Dec 16;17(12):e1009473. doi: 10.1371/journal.pgen.1009473 (PMC8769293; doi:10.1371/journal.pgen.1009473)
Supplement: S1 Appendix — (DOCX) [file pgen.1009473.s001.docx]

S1 Appendix.

Materials and Methods

*Elicitation of nec3 Phenotype*

The experiments were conducted using a plexiglass box with 60x30x30 cm (H/W/L) dimensions with two 7.5 cm holes on opposite sides of the box to allow for airflow. The ventilation holes were packed with cheesecloth to protect plants inside the box from pathogen spores or insects. The wildtype (WT) cv Bowman and *nec3-γ1* (Bowman background) seeds were planted and observed for 1 month. Two six-inch pots planted with two seedlings of each genotype were placed inside the isolation box in the greenhouse and two pots of the controls were maintained just outside the isolation box on the same greenhouse bench. The plants were watered as needed using distilled autoclaved water. Growth conditions inside the isolation box (sterile conditions) were 16-hour photoperiod at ~ 26^o^C +/- 3^o^C due to greenhouse effect of the plexiglass chamber and the normal greenhouse conditions outside of the isolation box were 16-hour photoperiod at ~22^o^C +/- 3^o^C. The control plants outside the box were either non-inoculated or inoculated with *B. sorokiniana* isolate ND85F using the procedure described below.

Following observation of the *nec3* phenotype on non-inoculated and *B. sorokiniana* isolate ND85F inoculated *nec3-γ1* plants in the greenhouse, leaves were analyzed to determine if an identifiable pathogen was present/colonizing the lesion sites. Following photographic documentation, the leaves containing lesions were removed, surface sterilized using 10% bleach for one minute, rinsed and plated on water agar plates and grown under light for 16 hours/day for 3-5 days. For the *B. sorokiniana* inoculated plants the *nec3* lesions were primarily colonized by *B. sorokiniana.,* However, the non-inoculated *nec3* plants had typical *nec3* lesions that were primarily colonized by *Blumeria graminis* the causal agent of the disease powdery mildew.

Pathogens that Induce *nec3* Phenotype

Ten WT Bowman and ten *nec3-γ1* plants were grown in a growth chamber with a 14 hours photoperiod at 22.5^o^C and 10 hours dark period at 19^o^C. The inoculum was produced by placing a PDA plug with *B. sorokiniana* isolate ND85F derived from infected leaves on a V8- potato dextrose agar (V8-PDA) plate followed by incubation at room temperature for ten days under constant light. The conidiospores were resuspended in sterile H_2_O using a sterile loop and adjusted to a concentration of ~2,000 spores/mL with 10ul of Tween20 in order to reduce spore clustering. When the secondary leaves were fully expanded approximately ten-day old seedling leaves were inoculated with *B. sorokiniana* isolate ND85F, the barley spot blotch pathogen, with an atomizer (campbell Hausfeld air paint sprayer, Dh580000av) with 50mL of conidiospores at ~2,000 spores/mL using 34.47 kPa of pressure. Once inoculated, the seedlings were placed in a dark mist chamber with misting every 12 minutes for 30s for 16 hours. After overnight misting, the plants were placed in the growth chamber and observed every day for the standard *nec3* phenotype to compare to the WT infection types and typical lesion development for 10 days (Fig 2).

Two forms of the net blotch pathogen, *Pyrenophora teres* f. *teres* and *P. teres* f. *maculata,* the causal agents of net form and spot form net blotch, respectively, were inoculated on the *nec3-γ1* and WT Bowman seedlings. The inoculum production and inoculation procedure used for *Pyrenothora teres* f. *teres* were performed as described in Shjerve et al., (2014) [1]. The inoculum production and inoculation procedure used for *Pyrenophora teres* f. *maculata* were performed as described in Neupane et al., 2015 [2]*.* After 7 days post-inoculation the secondary leaves were observed and scored for the standard *nec3* phenotype to compare to the WT infection types and typical net form net blotch and spot form net blotch lesions.

The *Blumeria graminis* inoculations were performed by gently tapping leaves with powdery mildew lesions above the experimental varieties in order to disperse spores from the infected leaves. Since *B. graminis* is an obligate biotroph, *B. graminis* infected barley leaves from the greenhouse were used as an inoculum source. Following spore application, the plants were left in the greenhouse (16 hour photoperiod at 24^o^C light and dark 16^o^C) until symptoms developed, which were compared between the WT infection types and the lesion that developed on the *nec3-γ1* mutant line (Fig 2).

*Pyrenophora tritici-repentis* was cultured on V8-PDA media by incubation in the dark for 5-6 days followed by a 24 hour incubation under light at room temperature and then 24 hours in the dark at 15^o^C [3]. The conidia were then harvested in sterilized distilled water (about 30 ml) by gently scratching the surface of the culture with a sterilized inoculation loop. Final spore concentration was adjusted to ~3000 spores/mL with 2 drops of Tween 20 per 100 mL of inoculum. The procedure described by Friesen and Faris (2004) was followed for the inoculation conditions and disease evaluation [4]. After a 7-day incubation period, the WT infection types with typical lesion were compared with the lesions that developed on the *nec3-γ1* mutant line (Fig 2).

*Parastagonospora nodorum* was grown on V8-PDA by spreading 300µl of pycnidial spores and incubated for 7 days under light at room temperature [5]. The spores were harvested by washing the plates with distilled water and adjusted to 10^6^ spores/mL with 2 drops of Tween 20 per 100 mL of inoculum [5]. The procedure described by Liu et al. (2004) was followed for inoculation conditions and disease evaluation. After a 7-day incubation period the WT infection types with highly resistant necrotic flecking were compared with the lesions that developed on the *nec3-γ1* mutant line (Fig 2).

*Cercospora beticola* was grown on V8-PDA media at 13^o^C under 24 hours of cool white light. The conidial suspensions were harvested in distilled water and adjusted to ~1.2 X 10^4^ spores/ mL with two drops of tween 20. Inoculation was performed as described by Einbeck, (1970) [6]. The WT and the *nec3-γ1* mutant line were observed for 35 days for lesion on the leaves (Fig 2).

Inoculations with *Puccinia graminis* f.sp. *tritici* races HKHJC (avirulent on cv Bowman containing the *Rpg1* stem rust resistance gene [7] and race QCCJB virulent on Bowman containing the *Rpg1* stem rust resistance gene [8] were performed after the primary leaves were fully expanded, approximately 10 day old seedlings. The inoculations were performed with *P. graminis* f.sp. *tritici* races HKHJC and QCCJB following the previously established methods described by Steffenson et al. [9]. Infection types were assessed 12 to 14 days post-inoculation using a 0-4 scale modified from the one developed for wheat by Stakman et al. [10]. The plants were also observed for the typical *nec3* phenotype from 1-14 days post inoculation (Fig 2).

*Xanthomonas translucens* pv *undulosa* strain BLS-LB10, originally cultured from a wheat plant in Lisbon, ND was grown on WBA plates for 3 days at 28^o^C and resuspended in sterile, distilled water at 0.2 OD [11,12]. Three *nec3-γ1* and three bowman wildtype seedlings were infiltrated with the inoculum using a syringe without a needle on the secondary leaves as described in Faris et al., 2010. Inoculated seedlings were then placed in a growth chamber under a 14 hours light at 22^o^C and 10 hours dark at 19^o^C cycle. The symptoms were observed and documented seven days post-infiltration (Fig 2).

## Infiltrations with DAMPs

1mL of the suspension (10-20k spores) was taken from the plates and added to 75-100mL of Fries media following Liu et al., 2004. The flasks containing Fries media and *B. sorokiniana* spores were incubated at 26^o^C in the dark for three days with shaking at 100rpm then placed in the dark at room temperature with continued shaking for an additional four days. Following 7 days of growth, the exudates were filtered with Miracloth and concentrated using a 15mL Microsep Advance Centrifugal Device with a 3kD size exclusion to concentrate the exudates ~8x.

DNA Extraction and Genotyping

DNA extraction of tertiary leaves was performed by homogenizing a 4 cm leaf section in 400µl of extraction buffer (200 mM Tris-HCl pH 7.5, 250 mM NaCl, 25 mM EDTA, 0.5% SDS). After homogenization with a disposable tissue grinder, 200ul of chloroform was added followed by vortexing for 10s and centrifugation at 16.1 rcf for 10 minutes. The supernatant (300ul) was transferred to a new tube and 200ul of chilled 99.7% isopropanol was added. The tubes were placed in 4^o^C for 10 minutes followed by centrifugation at 16.1 rcf for 10 minutes. The supernatant was poured off and the DNA pellet was rinsed with 75% EtOH. After rinsing and removal of excess EtOH, the DNA pellet was air dried for 15 minutes and resuspended in 50ul sterile water. For genotyping the standard parameters for the PCR reactions were: 94^o^C for 5 minutes, followed by 35 cycles of 94^o^C for 30 seconds, 62^o^C for 30 seconds, and 72^o^C for 60 seconds, with a final extension at 72^o^C for 7 minutes. The PCR reactions consisted of 1.25 units of NEB Standard Taq polymerase, forward and reverse primers (1.2 µM), NEB Standard Taq buffer (1x), dNTPs (200 µM), in 25 µL reactions. PCR products were visualized on a 1% agarose gel containing GelRed (Biotium, CA). The DNA bands were excised then purified using the E.Z.N.A. Cycle Pure Kit (Omega Bio-Tek, Norcross, GA) following the manufacturers standard protocol. The purified amplicons were directly sequenced using the amplicon specific forward primer (Genscript, NJ).

### Nec3 Map Development

The Bowman and Quest PCR amplicons for the SSR marker Bmag0807 had a nucleotide difference large enough to be separated and visualized on a 1.5% agarose gel while the GBM1212 amplicons had small enough differences that they were separated on a 12.5% polyacrylamide gel (Amresco, NY) for genotype scoring. The sequencing of the GBM1053 and GBM1423 alleles identified SNPs between cvs Bowman and Quest that were utilized to develop CAPS markers (S1 Table) using the restriction enzymes BsiHKAI and StyI (New England Biolabs, MA), respectively. The CAPS marker fragments were visualized and scored from 1% agarose gels stained with GelRed (Biotium, CA).

Exome Capture and Analysis

DNA was extracted from excised embryos of five germinated seeds of WT Bowman, WT Steptoe, nec3- γ1 (Bowman background), nec3.l (Steptoe background), and nec3.m (Steptoe background) mutants. DNA extractions were performed on mechanically lysed samples using the PowerPlant Pro DNA isolation kit (Qiagen, CA), following the protocol described by Solanki et al. (64). The quality of extracted DNA was checked by running a 1 µL aliquot of gDNA on a 1% agarose gel supplemented with GelRED (Biotium, CA) fluorescent nucleic acid dye. The DNAs with good integrity showing a high molecular weight band ~15-20 kb with minimal low molecular weight smearing was quantified on a Qubit fluorometer using the Qubit Broad Range DNA Quantification kit (Thermo Fisher Scientific, MA). Enzymatic DNA shearing was optimized to generate a range of desired fragment sizes from 250-450 bp with digestion reactions consisting of 1.5 µg of gDNA in a 20 µl reaction with NEB dsDNA Fragmentase enzyme, 1x Fragmentase reaction buffer and 10mM MgCl2 (New England Biolabs, Ipswich MA). The digested DNA was analyzed on the Agilent 2100 Bioanalyzer (Agilent Technologies, CA) using a DNA 1000 kit (Agilent Technologies, CA) following the manufacturer protocol for chip loading and data analysis for size distribution. The 25-minute enzymatic digestion was found to produce the optimal fragment size distribution ranging between 250-450 base pairs and were used to produce fragmented DNA libraries of WT Bowman and WT Steptoe, as well as the *nec3- γ1*, *nec3.l*, and *nec3.m* mutants.

Fragmented gDNA samples were used for exome capture using the Roche NimbleGen SeqCap EZ Developer probe pool barley exome design 120426_Barley_BEC_D04 with a total capture design size of 88.6 Mb. After exome capture, the KAPA HTP gDNA library preparation kit was used for Illumina sequencing library preparation. The standard manufacturer protocol was followed for library preparation using the KAPA HTP kit, except for size selection being performed on a Pippin Prep gel purification system (Sage Science, MA) with a 250-450 bp targeted size selection. The gDNA used to prepare the barcoded barley whole exome capture multiplexed library was developed according to the seqCAP EZ Library SR user guide 4.1 protocol. Quality and size distribution of the final capture library was determined using a bioanalyzer following the manufacturer’s guidelines. A Qubit fluorometer was used to quantify the library for final dilution and subsequent sequencing on an Illumina NextSeq flow cell generating 150 base pair, single end reads.

The *nec3-γ1*, *nec3.l*, and *nec3.m* mutants along with WT Bowman and WT Steptoe sequencing reads were parsed by their specific barcodes and quality scores of the raw reads were determined by FQC dashboard [13]. The Illumina reads were imported into CLC Genomics Workbench v8.0 in FASTQ format and trimmed for the presence of adapter sequences. Mutant and wildtype reads were aligned to the barley reference genome [14] using the BWA ‘mem’ algorithm with default settings [15]. The alignments were used to identify deleted regions utilizing two separate data analysis pipelines, where small deletions (less than 100 bp) were identified using SAMtools ‘mpileup’ with default settings [16]. The identified variants were filtered for a minimum read depth of 3 and a minimum individual genotype quality of 10 using VCFtools [17]. As fast neutron mutagenesis may induce large chromosomal deletions, sequencing coverage was calculated across all exome capture targets using BEDTools ‘genomecov’ to identify full gene deletions [18,19].

*RNAseq Analysis*

The average read length was 151bp and total reads obtained were analyzed for quality using FastQC v0.11.5 [20]. The quality trimmed reads were then imported into CLC Genome Workbench 8.0.3 for differential gene expression analysis (Qiagen Bioinformatics, CA). The reads of the 12 samples (the three biological replicates of WT Bowman and *nec3-γ1* non-inoculated and 72 hpi) were aligned using the barley concatenated reference high confidence and low confidence gene list provided from the barley IBSC IPK 2019 database of cv Morex [21]. Alignment was performed using mismatch cost 2, insertion and deletion cost 3, length, similarity fraction 0.9. The reads were aligned for both strand specificity and maximum number of hits for a read set at 10. Expression was calculated by normalization of reads for read depth and gene length using the RPKM (reads per kilobase million). Empirical analysis of Digital Gene Expression (EDGE) was performed to run “Exact test” for two group comparison [22]. FDR corrected P< 0.05 and EDGE ≥3 fold regulation values were used to get the differentially regulated genes between the non-inoculated and inoculated samples for both genotypes to create the final list of differentially regulated candidate genes.

*Exome Capture for Identification of Candidate Genes*

Sequences obtained from the barley exome capture array 120426_Barley_BEC_D04, were analyzed to identify potential deletions within the 149 high confidence candidate genes in the nec3 region based on the WGA Morex sequence released in 2019 [21]. A total of 11 genes in the nec3 region were not represented in the exome capture probe set, as shown in S3 Table. The 138 annotated high confidence genes (S2 Table) present in the exome probe set and captured in the nec3 region were analyzed in WT Bowman, WT Steptoe and the allelic mutants nec3-γ1, nec3.l and nec3.m. No deletions were observed in the exons of the 138 genes from the three independent mutants. To address the pitfall of the 11 uncharacterized gene missing in the exome capture, RNAseq analysis was carried out to further identify sequence that may have been missed by the exome capture analysis.

*In vitro functional characterization of Nec3 protein*

The partial Nec3 protein (Nec3∆26) was expressed in the X33 strain of Pichia pastoris with a c-terminal 6x His tag. The initial 26 amino acid encoded for the transmembrane domain, thus removed for the secreted protein expression. Secreted protein was purified by His-NTA column and analyzed by SDS-PAGE and western blot assay (S7 Fig). Purified Nec3∆26 protein’s possible T5H activity to convert Tryptamine to Serotonin was tested in vitro as described in Fujiwara et al. (2010) [23]. A 150 µl in vitro reaction was consisted of 20 mM potassium phosphate (pH 7.25), 50 pmol/ml recombinant Nec3 protein, NADPH reductase from rabbit liver, and 100 μM tryptamine. Reactions was initiated by adding 1mm NADPH and incubated at 30 °C for 30 min. Three controls, i.e., tryptamine (1 ng/µl) only, full reactions without Nec3, and without NADPH was carried out in parallel. After incubation samples were spiked with deuterated Serotonin as internal control, diluted 1:5 with ice cold methanol and centrifuged at maximum speed for 15 minutes to get the supernatant which was concentrated overnight in the speed vacuum. Final samples were subjected to LC-MS analysis on Agilent 6495 triple quadrupole system for Tryptophan to Serotonin conversion analysis.

**References**

1. Shjerve RA, Faris JD, Brueggeman RS, Yan C, Zhu Y, Koladia V, et al. Evaluation of a Pyrenophora teres f. teres mapping population reveals multiple independent interactions with a region of barley chromosome 6H. Fungal Genet Biol. 2014 Sep;70:104–112.

2. Neupane A, Tamang P, Brueggeman RS, Friesen TL. Evaluation of a barley core collection for spot form net blotch reaction reveals distinct genotype-specific pathogen virulence and host susceptibility. Phytopathology. 2015 Apr;105(4):509–517.

3. Lamari L, Bernier CC. Virulence of isolates of *Pyrenophora tritici-repentis* on 11 wheat cultivars and cytology of the differential host reactions. Canadian Journal of Plant Pathology. 1989 Sep;11(3):284–290.

4. Friesen TL, Faris JD. Molecular mapping of resistance to Pyrenophora tritici-repentis race 5 and sensitivity to Ptr ToxB in wheat. Theor Appl Genet. 2004 Aug;109(3):464–471.

5. Liu ZH, Friesen TL, Rasmussen JB, Ali S, Meinhardt SW, Faris JD. Quantitative Trait Loci Analysis and Mapping of Seedling Resistance to Stagonospora nodorum Leaf Blotch in Wheat. Phytopathology. 2004 Oct;94(10):1061–1067.

6. Einbeck KS. Cercospora. Kleinwanzlebener Saatzucht Ag Einbeck Rabbethge and Geisecke. 1970;

7. Brueggeman R, Rostoks N, Kudrna D, Kilian A, Han F, Chen J, et al. The barley stem rust-resistance gene Rpg1 is a novel disease-resistance gene with homology to receptor kinases. Proc Natl Acad Sci USA. 2002 Jul 9;99(14):9328–9333.

8. Nirmala J, Brueggeman R, Maier C, Clay C, Rostoks N, Kannangara CG, et al. Subcellular localization and functions of the barley stem rust resistance receptor-like serine/threonine-specific protein kinase Rpg1. Proc Natl Acad Sci USA. 2006 May 9;103(19):7518–7523.

9. Steffenson BJ, Jin Y, Brueggeman RS, Kleinhofs A, Sun Y. Resistance to stem rust race TTKSK maps to the rpg4/Rpg5 complex of chromosome 5H of barley. Phytopathology. 2009 Oct;99(10):1135–1141.

10. Stackman EC, Stewart DM, Loegering WQ. Identification of physiologic races of Puccinia graminis var. tritici. USDA. Agricultural Research Service E. 1962;617.

11. Duveiller E. The Bacterial Diseases of Wheat: Concepts and Methods of Disease Management. Duveiller E, editor. CIMMYT; 1997.

12. Adhikari TB, Gurung S, Hansen JM, Jackson EW, Bonman JM. Association mapping of quantitative trait loci in spring wheat landraces conferring resistance to bacterial leaf streak and spot blotch. The Plant Genome Journal. 2012;5(1):1.

13. Brown J, Pirrung M, McCue LA. FQC Dashboard: integrates FastQC results into a web-based, interactive, and extensible FASTQ quality control tool. Bioinformatics. 2017 Oct 1;33(19):3137–3139.

14. International Barley Genome Sequencing Consortium, Mayer KFX, Waugh R, Brown JWS, Schulman A, Langridge P, et al. A physical, genetic and functional sequence assembly of the barley genome. Nature. 2012 Nov 29;491(7426):711–716.

15. Li H, Durbin R. Fast and accurate long-read alignment with Burrows-Wheeler transform. Bioinformatics. 2010 Mar 1;26(5):589–595.

16. Li H, Handsaker B, Wysoker A, Fennell T, Ruan J, Homer N, et al. The Sequence Alignment/Map format and SAMtools. Bioinformatics. 2009 Aug 15;25(16):2078–2079.

17. Danecek P, Auton A, Abecasis G, Albers CA, Banks E, DePristo MA, et al. The variant call format and VCFtools. Bioinformatics. 2011 Aug 1;27(15):2156–2158.

18. Quinlan AR, Hall IM. BEDTools: a flexible suite of utilities for comparing genomic features. Bioinformatics. 2010 Mar 15;26(6):841–842.

19. Solanki S, Richards J, Ameen G, Wang X, Khan A, Ali H, et al. Characterization of genes required for both Rpg1 and rpg4-mediated wheat stem rust resistance in barley. BMC Genomics. 2019 Jun 14;20(1):495.

20. Andrews S. FastQC: a quality control tool for high throughput sequence data. 2010;

21. Monat C, Padmarasu S, Lux T, Wicker T, Gundlach H, Himmelbach A, et al. TRITEX: chromosome-scale sequence assembly of Triticeae genomes with open-source tools. Genome Biol. 2019 Dec 18;20(1):284.

22. Marioni JC, Mason CE, Mane SM, Stephens M, Gilad Y. RNA-seq: an assessment of technical reproducibility and comparison with gene expression arrays. Genome Res. 2008 Sep;18(9):1509–1517.

23. Fujiwara T, Maisonneuve S, Isshiki M, Mizutani M, Chen L, Wong HL, et al. Sekiguchi lesion gene encodes a cytochrome P450 monooxygenase that catalyzes conversion of tryptamine to serotonin in rice. J Biol Chem. 2010;285: 11308–11313. doi:10.1074/jbc.M109.091371
