# Supplementary figures and images for: Mutations in a barley cytochrome P450 gene enhances pathogen induced programmed cell death and cutin layer instability
Source: PLoS Genet. 2021 Dec 16;17(12):e1009473. doi: 10.1371/journal.pgen.1009473 (PMC8769293; doi:10.1371/journal.pgen.1009473)

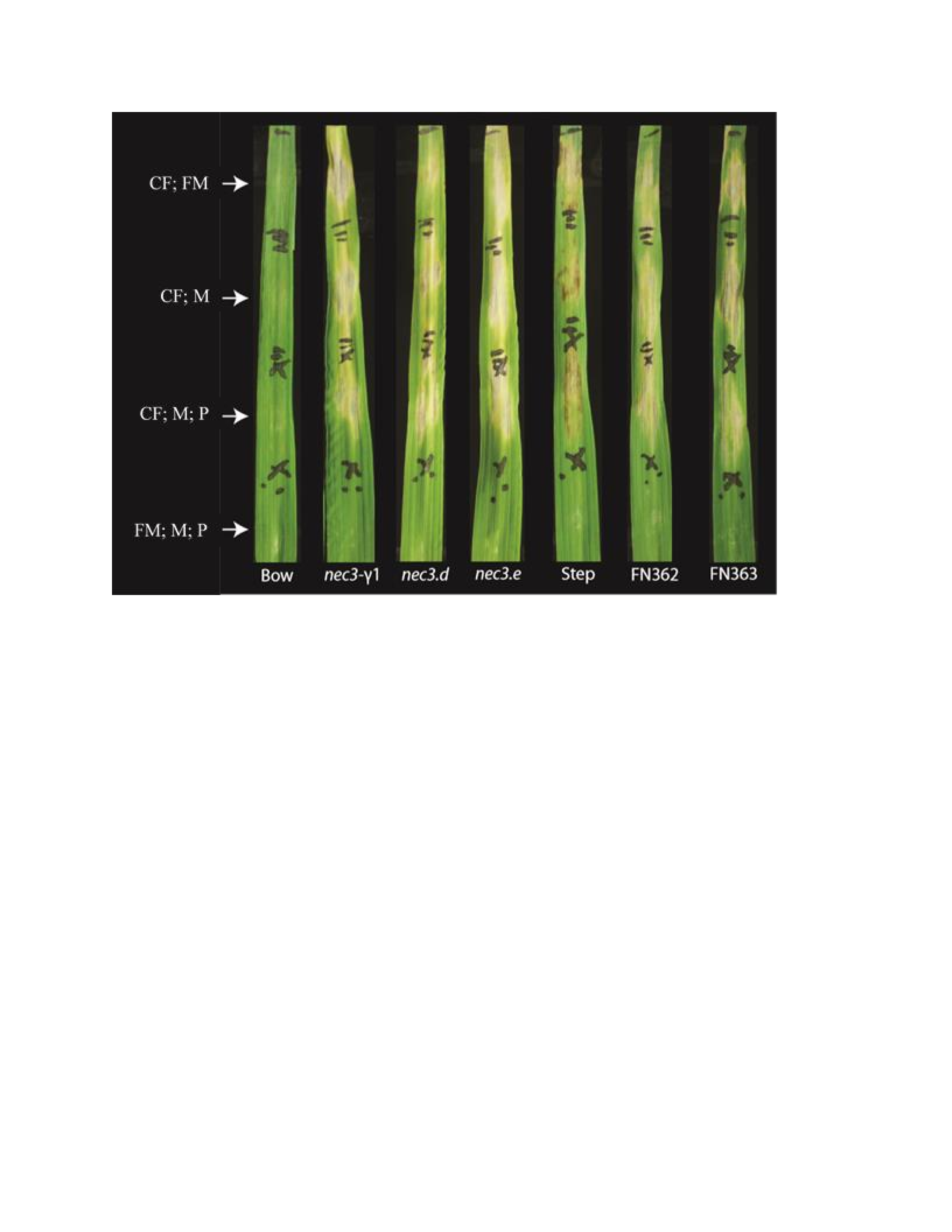

Supplement: S1 Fig — Infiltrations of secondary leaves of barley lines Bowman wt, nec3-γ1, nec3.d, nec3.e., Steptoe wt, nec3.l, and nec3.m (left to right) with Bipolaris sorokiniana isolate ND85F culture filtrates (CF). The treatments from top to bottom (indicated to the left) were culture filtrates (CF) with Fries media (FM); CF with MOPS buffer (M); CF with M and pronase (P); and the control containing FM, M and P. Infiltrations were performed at the two-leaf stage and documented 4 and 7 days after infiltration. Pictures shown were taken at 7 days post infiltration. (TIF) [file pgen.1009473.s006.tif]

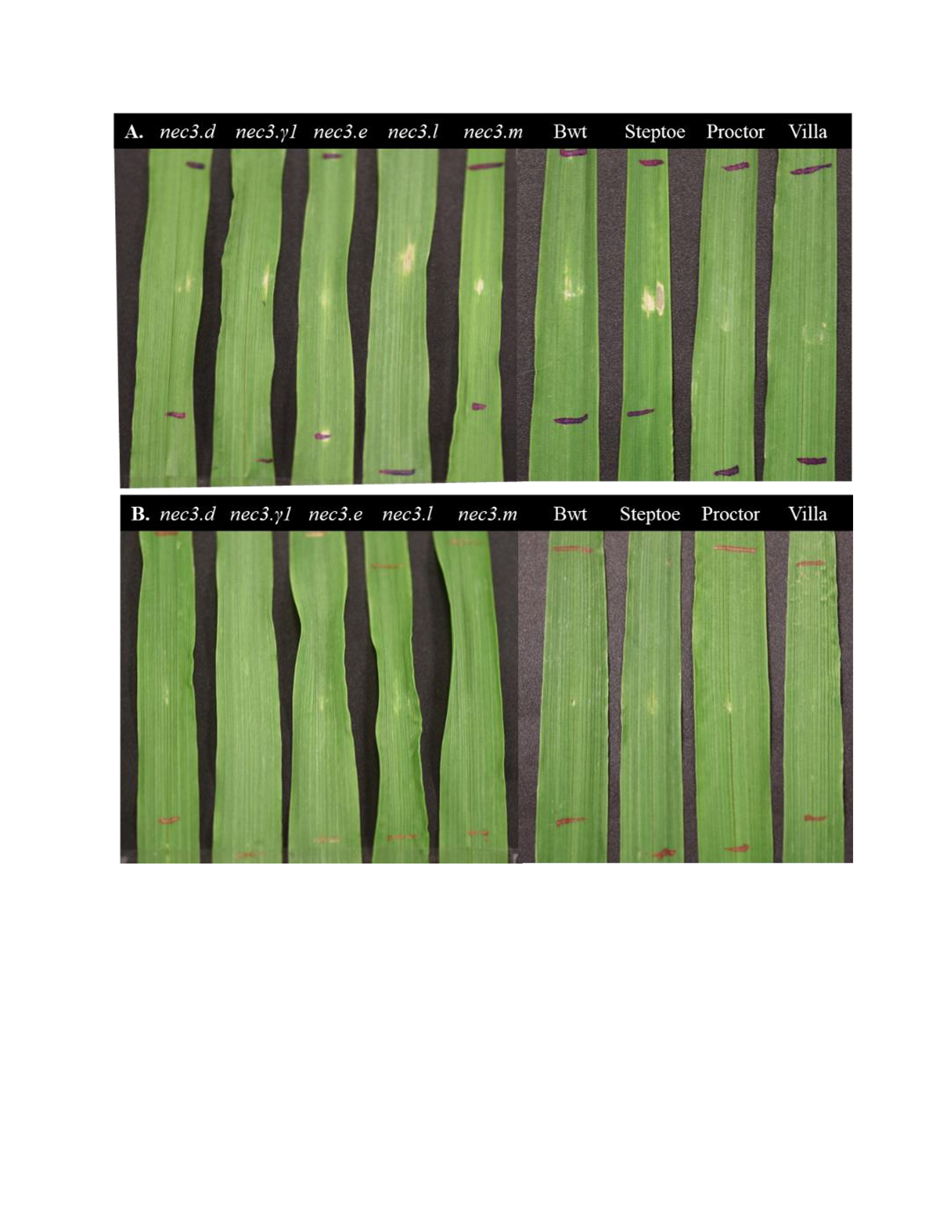

Supplement: S2 Fig — (A.) The panel shows the reaction to the chitin 2μg/ml infiltrations on the nec3 mutants from the left nec3.d, nec3.γ1, nec3.e, nec3.l and nec3.m, followed by the Bowman, Steptoe, Proctor, Villa wildtypes. (B.) The panel shows the reaction to the control buffer infiltrations on the nec3 mutants from the left nec3.d, nec3.γ1, nec3.e, nec3.l and nec3.m, followed by the Bowman, Steptoe, Proctor, Villa wildtypes. (TIF) [file pgen.1009473.s007.tif]

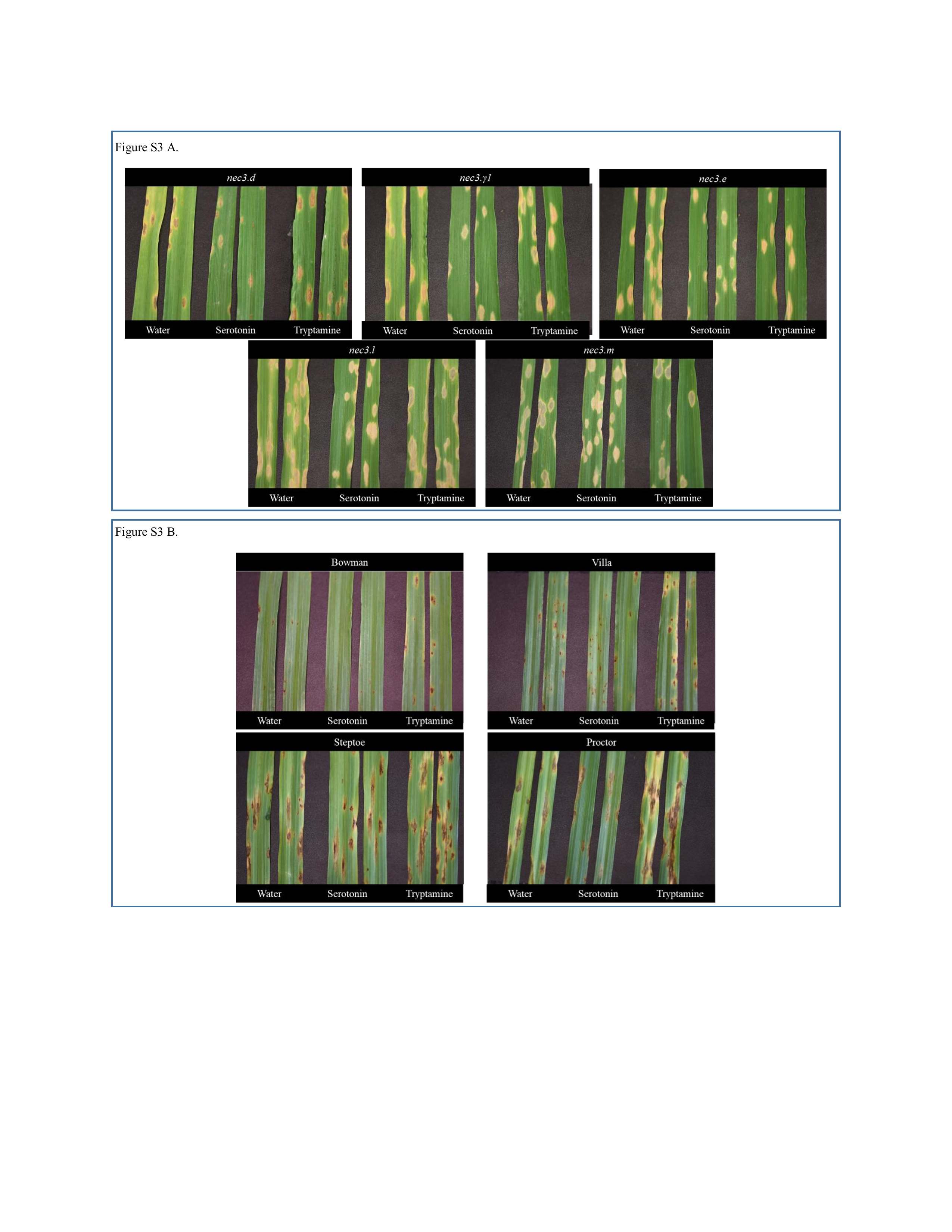

Supplement: S3 Fig — The nec3 phenotype induced by Bipolaris sorokiniana inoculation on the secondary leaf of the nec3 mutants (A.) and their respective wildtype barley plants (B.), where plants were supplied with 40 ml/pot of water; serotonin 150 μg/ml and Tryptamine 5mM on every alternate days starting from 5 days old seedling for a total of 10 root feedings. (TIF) [file pgen.1009473.s008.tif]

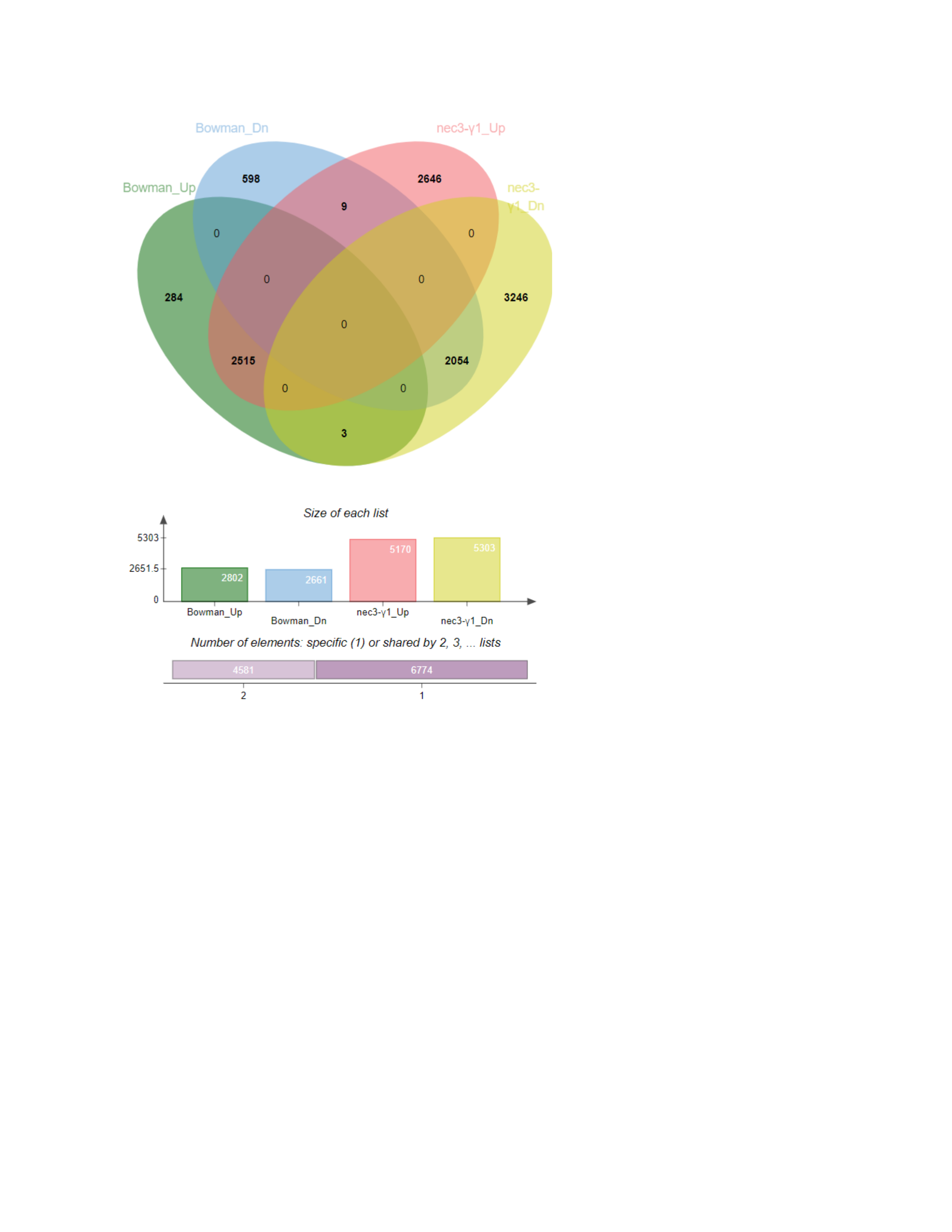

Supplement: S4 Fig — The venn-diagram represents the number of unique genes in a given class, where the blue and green oval represents Bowman downregulated and upregulated genes, respectively and the red and yellow oval represents nec3-γ1 mutant upregulated and downregulated genes, respectively (Top image). The bar graph shows the number of total genes present in each class Bowman and nec3-γ1 mutant up and down-regulated genes (Bottom image). (TIF) [file pgen.1009473.s009.tif]

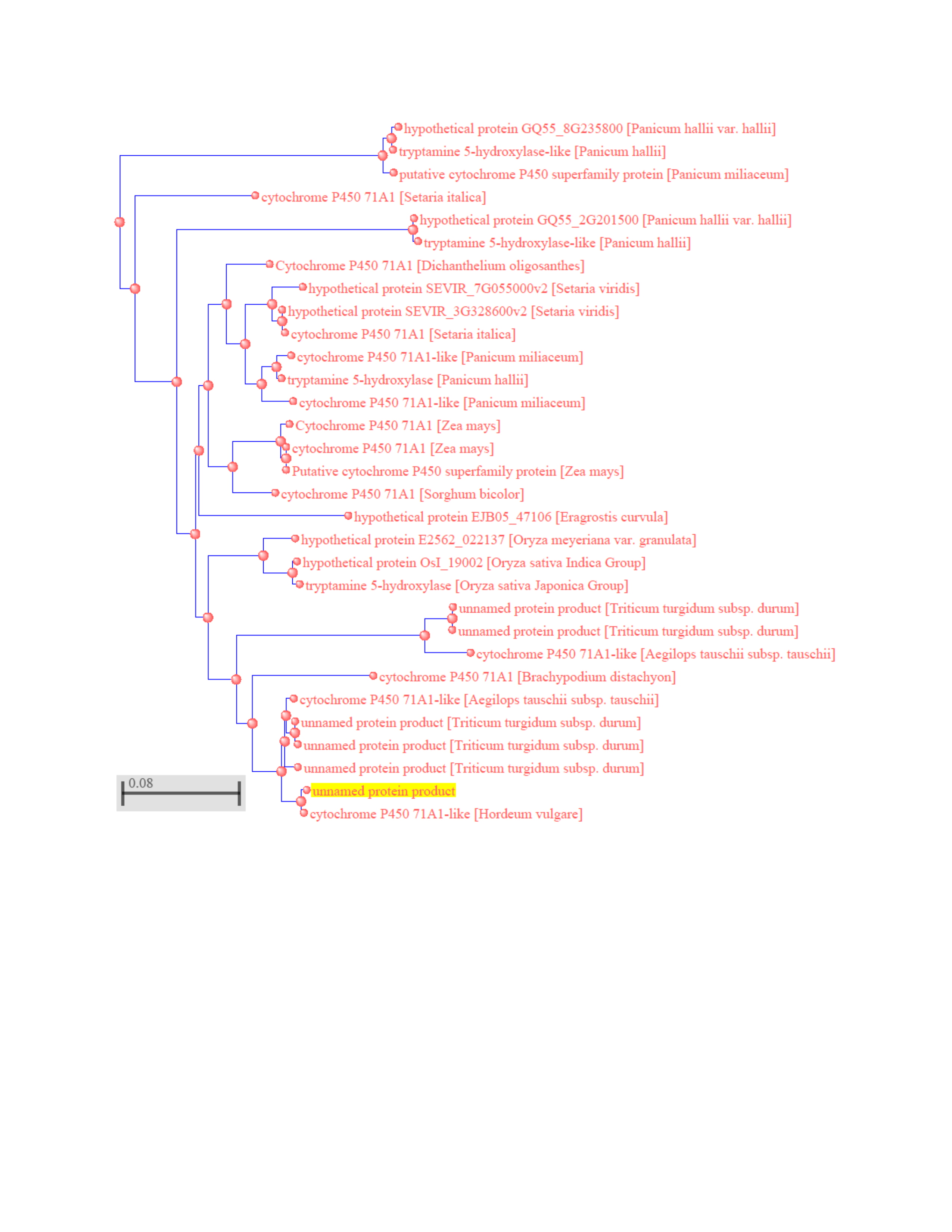

Supplement: S5 Fig — (TIF) [file pgen.1009473.s010.tif]

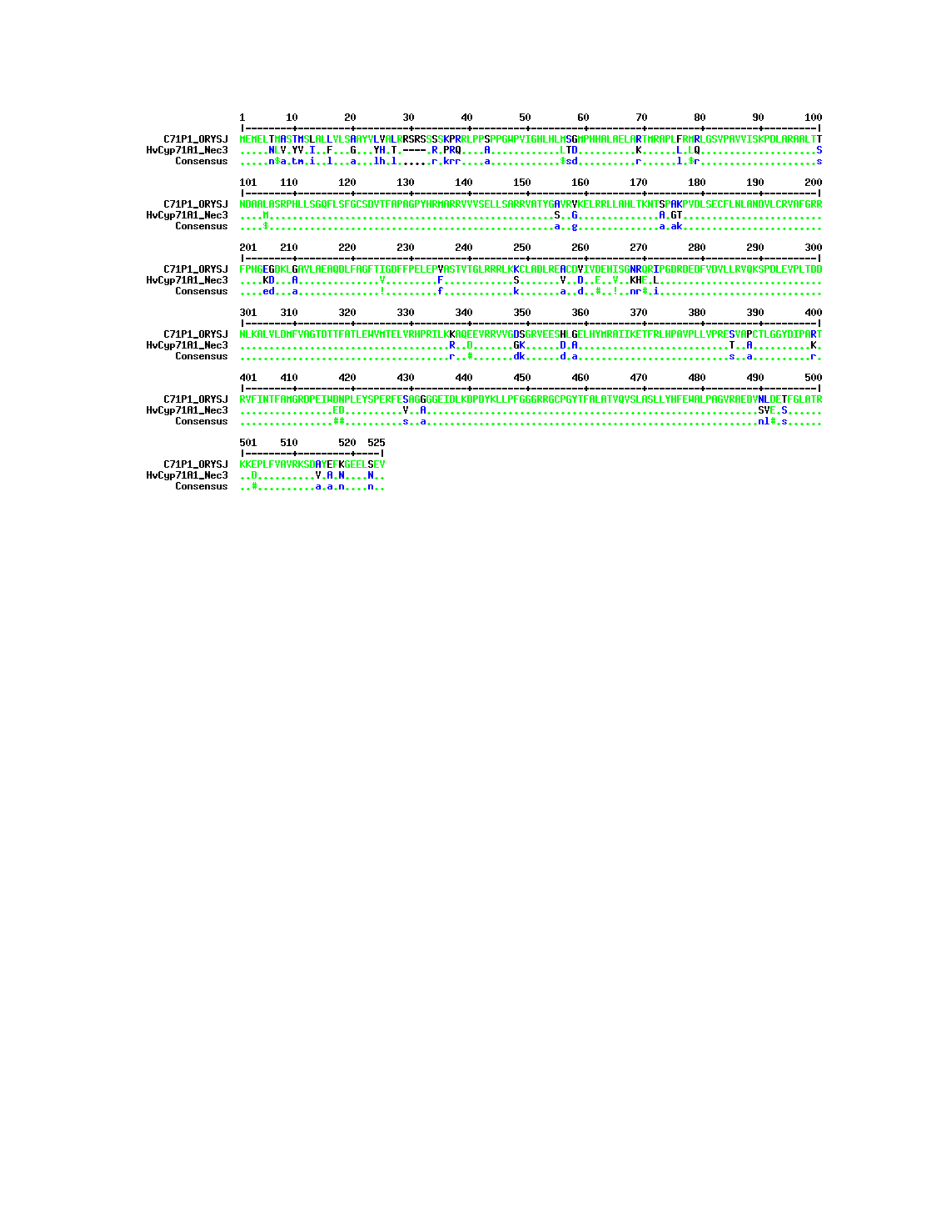

Supplement: S6 Fig — (TIF) [file pgen.1009473.s011.tif]

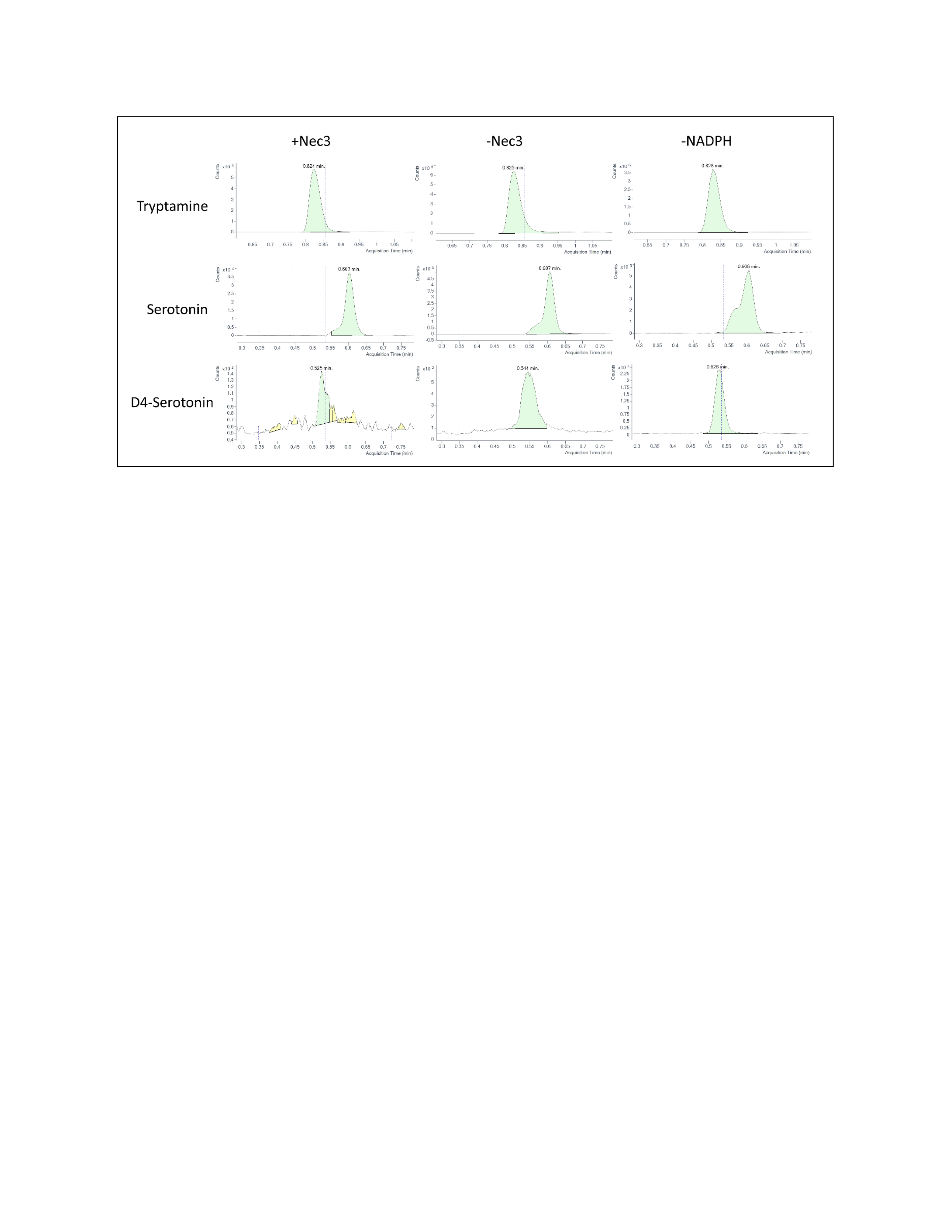

Supplement: S7 Fig — (TIF) [file pgen.1009473.s012.tif]
